# Machine learning-assisted rapid determination for traditional Chinese Medicine Constitution

**DOI:** 10.1186/s13020-024-00992-0

**Published:** 2024-09-15

**Authors:** Wen Sun, Minghua Bai, Ji Wang, Bei Wang, Yixing Liu, Qi Wang, Dongran Han

**Affiliations:** 1 School of Management, Beijing University of Chinese Medicine, Beijing, 100029 China; 2 School of Traditional Chinese Medicine/National Institute of TCM Constitution and Preventive Medicine, Beijing University of Chinese Medicine, Beijing, 100029 China; 3 School of Life Sciences, Beijing University of Chinese Medicine, Beisanhuan East Road No. 11, Chaoyang District, Beijing, 100029 China

**Keywords:** Automated machine learning (AutoML), Unsupervised machine learning, Constitution in Chinese Medicine Questionnaire (CCMQ), Tree-based Pipeline Optimization Tool (TPOT), Variable clustering (varclus)

## Abstract

The aim of this study was to develop a machine learning-assisted rapid determination methodology for traditional Chinese Medicine Constitution. Based on the Constitution in Chinese Medicine Questionnaire (CCMQ), the most applied diagnostic instrument for assessing individuals’ constitutions, we employed automated supervised machine learning algorithms (i.e., Tree-based Pipeline Optimization Tool; TPOT) on all the possible item combinations for each subscale and an unsupervised machine learning algorithm (i.e., variable clustering; varclus) on the whole scale to select items that can best predict body constitution (BC) classifications or BC scores. By utilizing subsets of items selected based on TPOT and corresponding machine learning algorithms, the accuracies of BC classifications prediction ranged from 0.819 to 0.936, with the root mean square errors of BC scores prediction stabilizing between 6.241 and 9.877. Overall, the results suggested that the automated machine learning algorithms performed better than the varclus algorithm for item selection. Additionally, based on an automated machine learning item selection procedure, we provided the top three ranked item combinations with each possible subscale length, along with their corresponding algorithms for predicting BC classification and severity. This approach could accommodate the needs of different practitioners in traditional Chinese medicine for rapid constitution determination.

**Supplementary Information:**

The online version contains supplementary material available at 10.1186/s13020-024-00992-0.

## Introduction

The Yellow Emperor's Classic of Medicine introduced the concept of “weibing,” which refers to the state preceding the onset of disease, essentially describing a condition of subhealth. Within conventional medicine paradigms, the state between health and disease is referred to as the “third state”. However, the scope of the “third state” is broad, and its mechanisms are unclear, which complicates targeted intervention strategies. In traditional Chinese medicine (TCM), there are systematic theories and intervention methods specifically designed to address subhealth issues. One of the common theoretical frameworks in TCM for subhealth is described using body constitution (BC), which is relatively stable across an individual’s lifespan.

BC arises from a combination of inherent genetic factors (e.g., race, family genetics, prenatal development, etc.) and acquired traits (e.g., dietary nutrition, lifestyle habits, mental state, disease damage, drug treatment, etc.). BC manifests as an individual’s physical morphology, physiological functions, psychological conditions, and interactions with environmental factors [1, 2]. Wang systematically analyzed the theoretical origins, formation, and development of body constitution theory based on 108 ancient Chinese traditional classic works and 168 modern documents. Combining current clinical research, Wang identified nine constitutional types: Gentleness Constitution (GTC: calm mentality, strong adaptability to the environment and resilience to illness), Qi-deficiency Constitution (QDC: shortness of breath and low energy), Yang-deficiency Constitution (YaDC: low tolerance to cold weather), Yin-deficiency Constitution (YiDC: insufficient body fluid), Phlegm-dampness Constitution (PDC: tendency to have abdominal obesity), Damp-heat Constitution (DHC: tendency of excessive humidity, overheating, sweating of hands and feet, yellow urine, and loose stools), Blood-stasis Constitution (BSC: tendency towards increased blood viscosity), Qi-stagnation Constitution (QSC: tendency to have depression, anxiety and chest pain), and Special-diathesis Constitution (SDC: tendency to have allergy) [1]. BC is determined by people’s observed characteristics (e.g., physiological, psychological, and reactive states) and their connections with the nature, occurrence, development, and outcome of different diseases [3]. Thus, the identification of BC may aid in understanding the pathological mechanisms of certain diseases, guiding therapeutic interventions and prognostic evaluations [4, 5].

TCM practitioners typically assess a patient's constitution through four examinations: observing, listening and smelling, questioning, and palpating. The accuracy of this approach largely depends on practitioners' expertise and experience. To standardize the BC type identification procedure, Wang developed the Constitution in Chinese Medicine Questionnaire (CCMQ), which quantitatively measures the extent to which an individual exhibits a specific BC [6]. The CCMQ has been widely used in clinical research and practice. Furthermore, it has been translated into several languages, including English, Korean, Japanese, and Vietnamese, enhancing its accessibility and applicability across different cultural contexts [7–10].

The CCMQ and its simplified versions have demonstrated robust reliability and validity in assessing body constitutions within the field of TCM [11–14]. However, scoring methods for such questionnaires generally rely on linear addition of items within a subscale, a process that fails to capture the nonlinear relationships inherent in TCM [15]. Moreover, even when a linear relationship is assumed, these traditional scoring methods often overlook the need to account for the relative weights of different items. To further improve the efficiency of the determination of BC classifications and scores, this study introduces machine learning-assisted methods for their rapid determination. These methods enable automatic classifications of BC and calculation of BC scores using selected subsets of CCMQ items. Utilizing both linear and nonlinear algorithms, machine learning-based approaches incorporate the relative weights of items, thereby refining the scoring and classification processes for BC types.

Machine learning methodologies can be broadly categorized into two primary types: supervised and unsupervised machine learning. During the process of supervised machine learning-assisted rapid determination, items are utilized as predictors, and each BC classification or score is used as the target to construct predictive models. A subset of core items that contribute most to the predicted outcome are chosen in the training process, and then they are used as predictors to obtain the outcome (i.e., BC classification or score) in the testing process. Common supervised machine learning algorithms for item selection include support vector machines, elastic net, extreme gradient boosting, gradient boosting, k-nearest neighbors, random forests, and extremely randomized trees. These algorithms have been proven effective in various studies [16, 17] and are considered potential supervised learning options in this study. In contrast, unsupervised machine learning does not involve predefined targets but instead aims to discover intrinsic data patterns and groupings based on the relationships among all items, potentially transcending the limitations of predefined dimensions. Using unsupervised methods, the most representative items for inherent groupings were retained. These items are subsequently utilized as features in supervised machine learning algorithms to predict BC classifications and scores. The dimensional structure and item formulation of the CCMQ are primarily designed based on the experiential insights of experts in TCM, lacking consistent structural validity supported by empirical data. As mentioned previously, supervised and unsupervised machine learning algorithms each offer distinct advantages in analyzing the relationships between the CCMQ dimensions and items. Supervised algorithms operate on the premise of acknowledging existing dimensions, while unsupervised algorithms do not. In the process of predicting BC classifications or scores with a limited number of items, comparing the selections made by these two approaches can help identify consistencies and discrepancies in item choice, which further reveals items that are crucial for the stability of the CCMQ dimensions.

In summary, this study illustrated the application of various machine learning methods to rapidly determine BC classifications and calculate BC scores. Specifically, we utilized an automated machine learning algorithms (AutoML) known as the Tree-based Pipeline Optimization Tool (TPOT), as well as unsupervised machine learning through variable clustering analysis (varclus). Using machine learning methods (i.e., model selection and parameter tuning), the predicted BC classifications or scores from a subset of core items are expected to be highly correlated with the original classifications or scores; thus, a subset of core items can be used instead of the whole set to improve the test efficacy of the CCMQ.

## Materials and methods

### Data source and collection

Between August 26, 2015, and October 12, 2017, a survey was conducted via a web-based platform using the CCMQ to assess the BC of individuals aged 15–64 across China. A total of 94,718 questionnaires were collected. However, 3573 responses were discarded due to fictitious or inconsistent answers, yielding 91,145 valid questionnaires for analysis.

#### Measurement instrument

This study employed the 60-item adult version of the CCMQ developed by Wang et al. (2006). The questionnaire is organized into nine subscales, each containing 6–8 items. Questions are presented in a question format (e.g., “Were you energetic?”) Responses are measured using a five-point Likert scale ranging from “1. Not at all” to “5. Very much.” Specific items associated with the GTC subscale include the following: “(2) Did you become tired easily?”, “(7) Was your voice weak when talking?”, “(8) Did you feel in low spirits and depressed?”, “(21) Did you feel more vulnerable to the cold than others (winter coldness, air conditioners, fans, etc.)?”, “(54) Did you easily experience insomnia?”, and “(27) Did you forget things easily?” are reverse scored. In contrast, these items are positively scored in other BCs as well as the remaining items. The formula for calculating the converted score is as follows:

$$\text{Converted Score}= \frac{\sum \text{Raw Score}-\text{Number of Items}}{\text{Number of Items}\times 4}\times 100$$

BC types were classified based on the criteria established in the “Classification and Determination of Chinese Medicine Constitution” [18]^.^ Specifically, the criterion for identifying GTC requires a minimum conversion score of 60, whereas the conversion scores for the remaining biased BCs are less than 30. For the eight biased BCs, a converted score above 40 indicates their presence, and scores ranging from 30 to 40 suggest a predisposition toward a specific constitution.

In this study, the Cronbach’s alpha coefficients for the subscales were as follows: GTC at 0.664, QDC at 0.742, YaDC at 0.778, YiDC at 0.673, PDC at 0.706, DHC at 0.649, BSC at 0.660, QSC at 0.775, and SDC at 0.721.

### Methods

#### Supervised machine learning

AutoML was employed to select the optimal supervised machine learning algorithm for BC classifications and scores, facilitating the implementation of traditional machine learning model design strategies in an automated, data-driven manner. The Tree-based Pipeline Optimization Tool (TPOT), a prevalent AutoML algorithm, automatically designs and optimizes machine learning pipelines for specific problem domains without human intervention [19]. TPOT primarily comprises two components: model construction via genetic programming and optimal model selection using Pareto efficiency. First, predictive models were built using all possible CCMQ item combinations as predictor variables, ranging from combinations with single item to all items, with the BC classifications and scores from the original subscales as the predicted outcomes. During the model construction process, new pipeline configurations are generated through crossover and mutation operations within the genetic algorithm. However, a challenge arises because maximizing model predictive performance (the model with the highest accuracy for classification and the model with the smallest mean squared error (MSE) for regression) often results in increased model complexity. By leveraging genetic programming in conjunction with Pareto optimality, the model selected by TPOT effectively balances predictive performance against complexity. Based on the model selected by the TPOT, the item combination that demonstrated the best performance across all item combinations with the same number of items was selected using the area under the curve (AUC) or R-squared (R^2^). Then, given the best item combinations for a specific number of items, we may need to further determine the most appropriate number of items for predicting BC classifications or scores. With this aim, we calculated the AUC or R^2^ improvement for the predictive performance of an item combination with at least two items over the item combination with one fewer item in predicting the original BC classifications or scores. In this study, the item combinations that show obvious improvements in AUC and R^2^ are recommended for rapidly assessing BC classifications and scores. When the improvement progresses steadily, it is recommended to use a threshold of 0.8 for AUC and R^2^, which are generally considered indicators of excellent predictive performance [20, 21], to determine the minimum number of items to be retained when predicting BC classifications or scores. Finally, the predictive performance will be assessed by AUC, accuracy, F1 score, R^2^, root mean square error (RMSE) and mean absolute percentage error (MAPE).

#### Unsupervised machine learning

Variable clustering analysis (varclus) was used in this study as the unsupervised machine learning method, which groups similar items into clusters. Each cluster can be represented by a single item. This approach reduces data complexity while ensuring interpretability. Based on the clusters, representative items are selected, and the scale items are simplified accordingly.

**Fig. 1:**
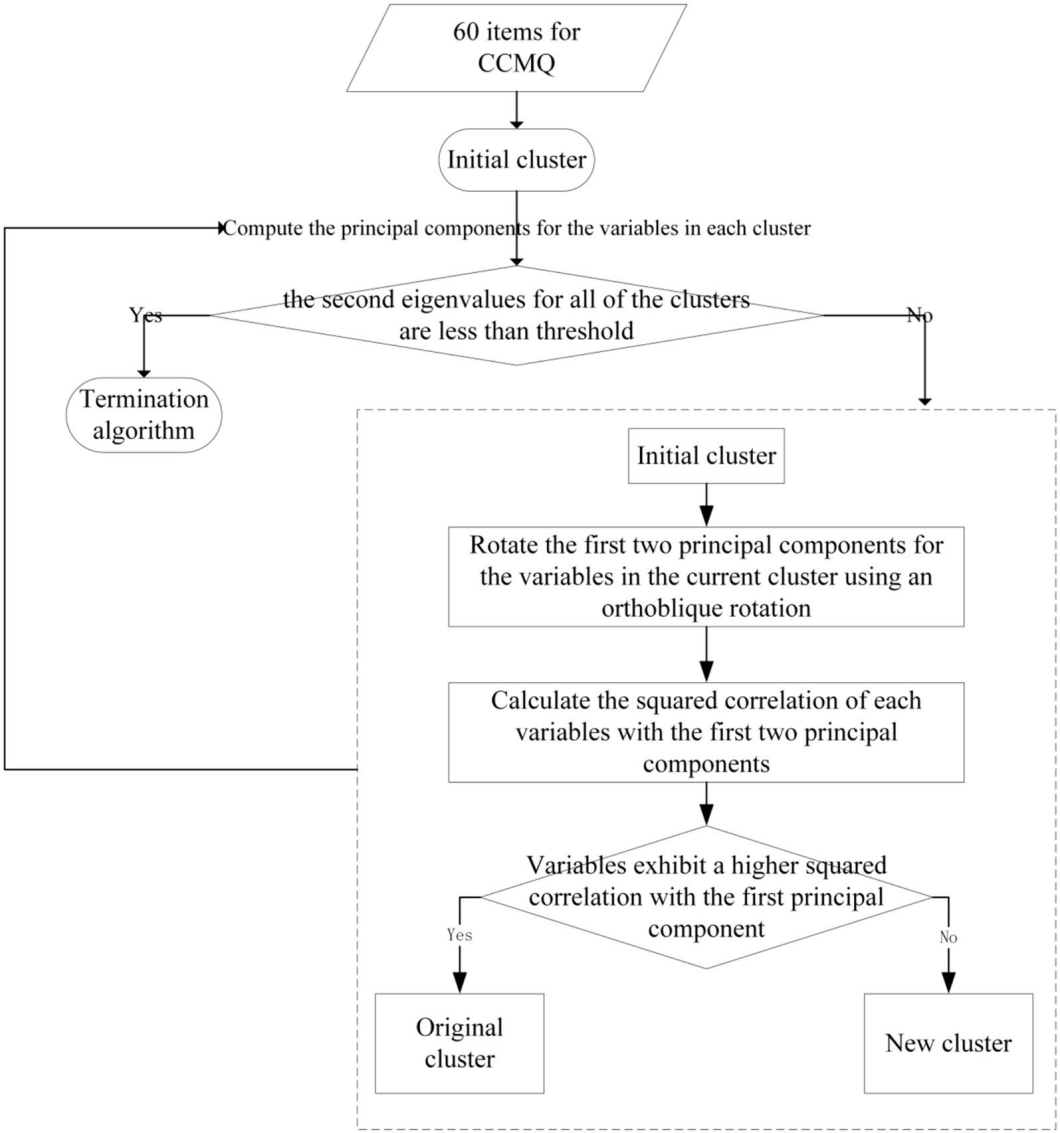
Clustering process

First, the 60 items of the CCMQ were normalized to form a single cluster. This cluster was then iteratively segmented, continuing until the second eigenvalue within a cluster did not exceed the threshold (shown in Fig. 1). In this study, the thresholds were dynamically adjusted to capture clusters of all possible quantities (i.e., 1–60). Next, representative items were selected from each cluster. The selection of representative items is based on the following formula:

$$\text{r}=\frac{{\uplambda }_{\text{ij}}}{{\uplambda }_{\text{j}}^{\text{max},\text{other}}}$$

${\uplambda }_{\text{ij}}$ represents the loading of the jth variable within the ith cluster on the first principal component. ${\uplambda }_{\text{j}}^{\text{max},\text{other}}$ represents the jth variable’s maximum loading on the first principal component of all other clusters, excluding the cluster to which it belongs. The item with the highest ratio is chosen as the representative item for a given cluster.

In other studies, the number of principal components is typically determined using a cumulative variance contribution rate of 70–90% [22]. In this study, we adopt the average variance contribution rate at a median level within this range to determine the number of clusters. We selected the least number of clusters based on the criterion that the average within-cluster variance explained by the representative item is 80%. The items selected by varclus were used to predict BC classifications or scores by selecting the appropriate supervised machine learning methods using the TPOT algorithms, and their predictive capacities were evaluated using AUC, accuracy, and F1 score (or R^2^, RMSE, and MAPE).

## Results

### Distribution of scores among different BCs

**Fig. 2:**
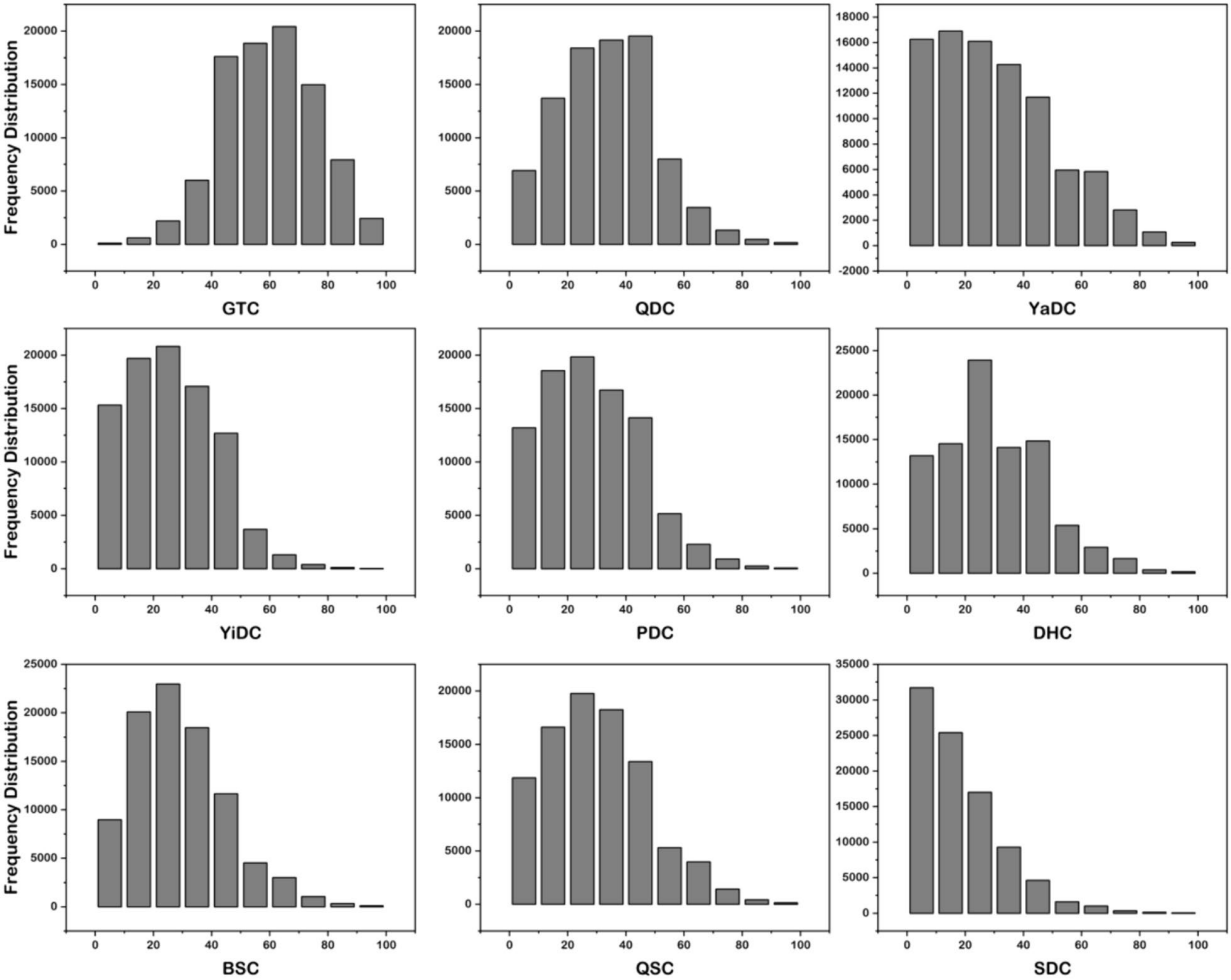
Distribution of scores for the BCs

Figure 2 shows that the scores for the eight biased BCs exhibit a pronounced left-skewed distribution. Among the seven biased BCs (i.e., QDC, YaDC, YiDC, PDC, DHC, BSC, and QSC), there was a greater number of individuals in the 20–50 score range.

Only 13.66% of the population exhibited GTC, 36.15% were classified as QDC, 30.34% as YaDC, 20.02% as YiDC, 25.07% as PDC, 27.83% as DHC, 22.66% as BSC, 27.08% as QSC, and 8.51% as SDC. It should be noted that some individuals may exhibit multiple biased BCs simultaneously.

### Item selection based on automated machine learning

**Fig. 3:**
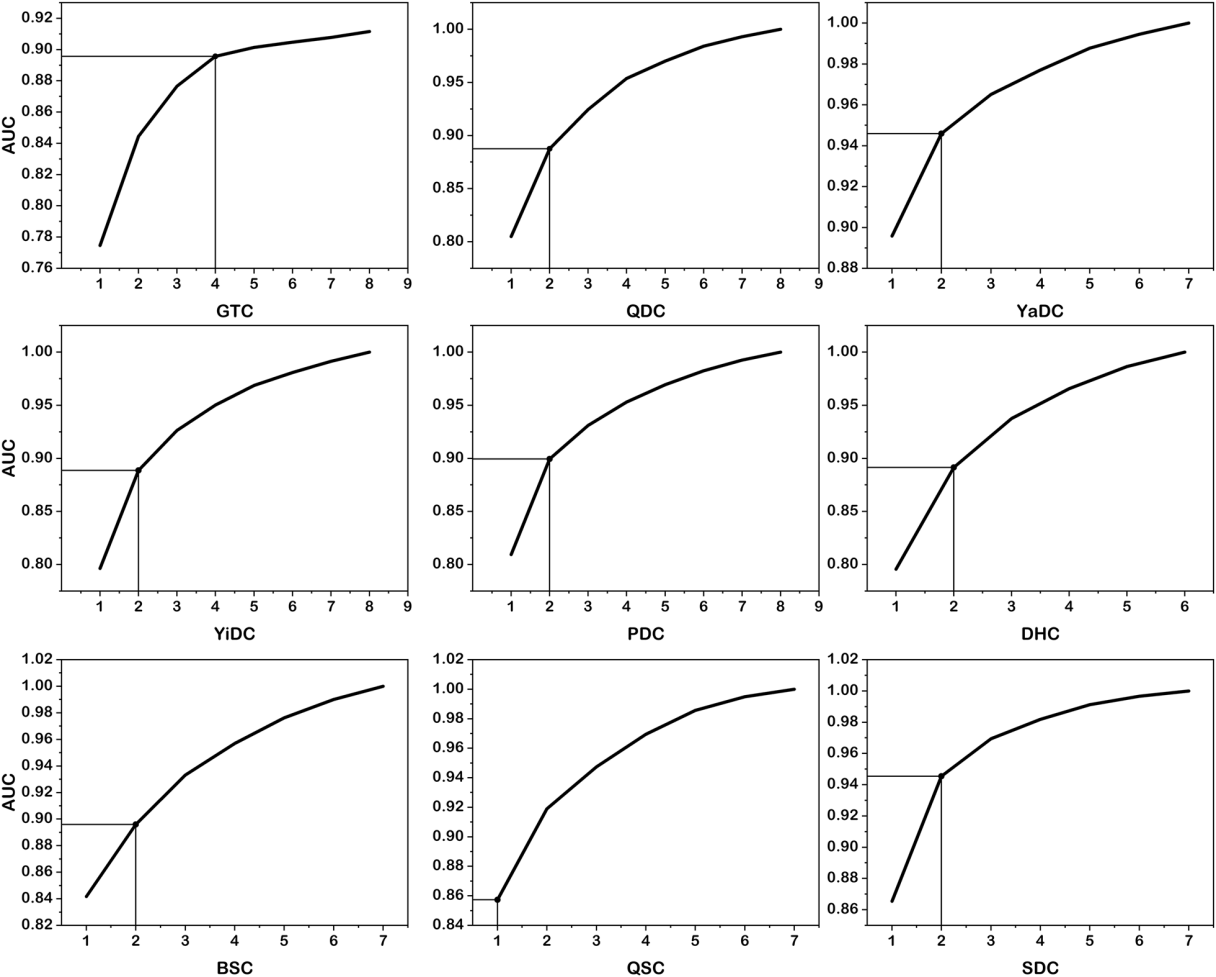
AUC for different item combinations. Note. For the GTC, QDC, YaDC, YiDC, PDC, DHC, BSC and SDC, elbow points were selected where the item combination maximized the improvement in the AUC. For QSC, the curve was relatively smooth, so we selected the item combination with the fewest number of items when the AUC exceeded 0.8

The optimal performance of the supervised machine learning item selection models, utilizing BC classifications as the dependent variable across various item combinations, is presented in Table 1, with the corresponding algorithms detailed therein. Figure 3 illustrates the improvement in the AUC for the optimal item combinations for predicting each BC classification.

**Table 1:** The optimal item combinations for BC classifications as the dependent variable and their corresponding algorithms

	GTC	QDC	YaDC	YiDC	PDC	DHC	BSC	QSC	SDC
1 item	2	3	19	20	49	60	40	9	30
	GaussianNB	MLPClassifier	MLPClassifier	MLPClassifier	MLPClassifier	MLPClassifier	MLPClassifier	GaussianNB	GaussianNB
			GaussianNB						
2 items	2, 21	3, 6	19, 52	20, 35	49, 50	39, 59	40, 43	9, 14	24, 31
	XGBClassifier	XGBClassifier	XGBClassifier	XGBClassifier	MLPClassifier	ExtratreesClassifier	MLPClassifier	XGBClassifier	GradientBoostingClassifier
3 items	2, 8, 21	3, 5, 7	18, 19, 52	20, 35, 44	15, 49, 50	39, 56, 60	37, 40, 43	9, 10, 14	24, 30, 31
	PolynomalFeatures	XGBClassifier	XGBClassifier	MLPClassifier	RobustScale	StandardScaler	MLPClassifier	ExtratreesClassifier	FectureUnion
	XGBClassifier				MLPClassifier	MaxAbsScaler			XGBClassifier
						MLPClassifier			
4 items	2, 8, 21, 27	3, 5, 7, 26	17, 18, 19, 52	16, 35, 38, 44	13, 28, 49, 50	39, 48, 56, 60	37, 40, 43, 45	9, 10, 12, 47	24, 30, 31, 34
	RobustScaler	RobustScaler	XGBClassifier	StandardScaler	GradientBoostingClassifier	MLPClassifier	MLPClassifier	GaussianNB	MLPClassifier
	XGBClassifier	MLPClassifier	MLPClassifier	MLPClassifier				MLPClassifier	
5 items	2, 7, 8, 21, 27	3, 5, 6, 22, 26	17, 18, 21, 22, 52	16, 38, 44, 46, 57	13, 28, 50, 51, 58	39, 41, 48, 56, 60	36, 37, 40, 43, 45	9, 10, 12, 14, 47	24, 25, 30, 31, 34
	MLPClassifier	MinMaxScaler	RobustScaler	MLPClassifier	RobustScaler	GaussianNB	MLPClassifier	RobustScaler	XGBClassifier
		MLPClassifier	MLPClassifier		MLPClassifier	MLPClassifier		MLPClassifier	
6 items	2, 7, 8, 21, 27, 54	3, 5, 6, 7, 22, 26	17, 18, 21, 22, 52, 55	16, 20, 29, 44, 46, 57	13, 28, 49, 50, 51, 58	39, 41, 48, 56, 59, 60	33, 36, 37, 40, 43, 45	9, 10, 11, 12, 14, 47	24, 25, 30, 31, 32, 34
	MLPClassifier	ZeroCount	RobustScaler	ZeroCount	MLPClassifier	MLPClassifier	RobustScaler	StandardScaler	RobustScaler
		MLPClassifier	MLPClassifier	MLPClassifier			MLPClassifier	MLPClassifier	GaussianNB
									MLPClassifier
7 items	2, 7, 8, 21, 27, 53, 54	3, 4, 5, 6, 7, 22, 26	17, 18, 19, 21, 22, 52, 55	16, 29, 35, 38, 44, 46, 57	13, 28, 42, 49, 50, 51, 58		33, 36, 37, 40, 43, 27, 45	8, 9, 10, 11, 12, 14, 47	23, 24, 25, 30, 31, 32, 34
	MLPClassifier	GaussianNB	MLPClassifier	RobustScale	RobustScale		MLPClassifier	MLPClassifier	LinearSVC
		StandardScaler		MLPClassifier	MLPClassifier				
		MLPClassifier							
8 items	1, 2, 7, 8, 21, 27, 53, 54	2, 3, 4, 5, 6, 7, 22, 26		16, 20, 29, 35, 38, 44, 46, 57	13, 15, 28, 42, 49, 50, 51, 58				
	ZeroCount	MLPClassifier		MLPClassifier	LinearSVC				
	MLPClassifier								

The specific meanings represented by the entries are found in the supplementary materials

For all models except the QSC model, the AUC plots revealed elbow points for either two or four items: GTC: item 2 (i.e.,tiredness; abbreviated form throughout; items, questions, and question abbreviations are provided in Table S1), item 8 (i.e., depression), item 21 (i.e., cold intolerance), item 27 (i.e., forgetfulness); QDC: item 3 (i.e., breathlessness), item 6 (i.e., quietude); YaDC: item 19 (i.e., cold aversion), item 52 (i.e., cold sensitivity); YiDC: item 20 (i.e., localized hotness), item 35 (i.e., dryness); PDC: item 49 (i.e., sticky mouth), item 50 (i.e., flabby abdomen); DHC: item 39 (i.e., oily skin), item 59 (i.e., urethral heat); BSC: item 40 (i.e., hyperpigmentation), item 43 (i.e., dark circles); and SDC: item 24 (i.e., chronic rhinitis), item 31 (i.e., urticaria). Remarkably, the QSC model achieved the predefined threshold of AUC = 0.8 with the inclusion of only one item (item 9: anxiety). The selected items achieved AUC values ranging from 0.857 to 0.946 (shown in Table 3). In general, predictive models are considered excellent when their AUC values fall between 0.8 and 0.9 and outstanding when they exceed 0.9 [21]; therefore, all these models demonstrated excellent predictive performance, with some even reaching outstanding levels. Additionally, the accuracy and F1 scores of these models' predictions were calculated, with accuracy values ranging from 0.819 to 0.936 and F1 scores ranging from 0.417 to 0.807.

**Fig. 4:**
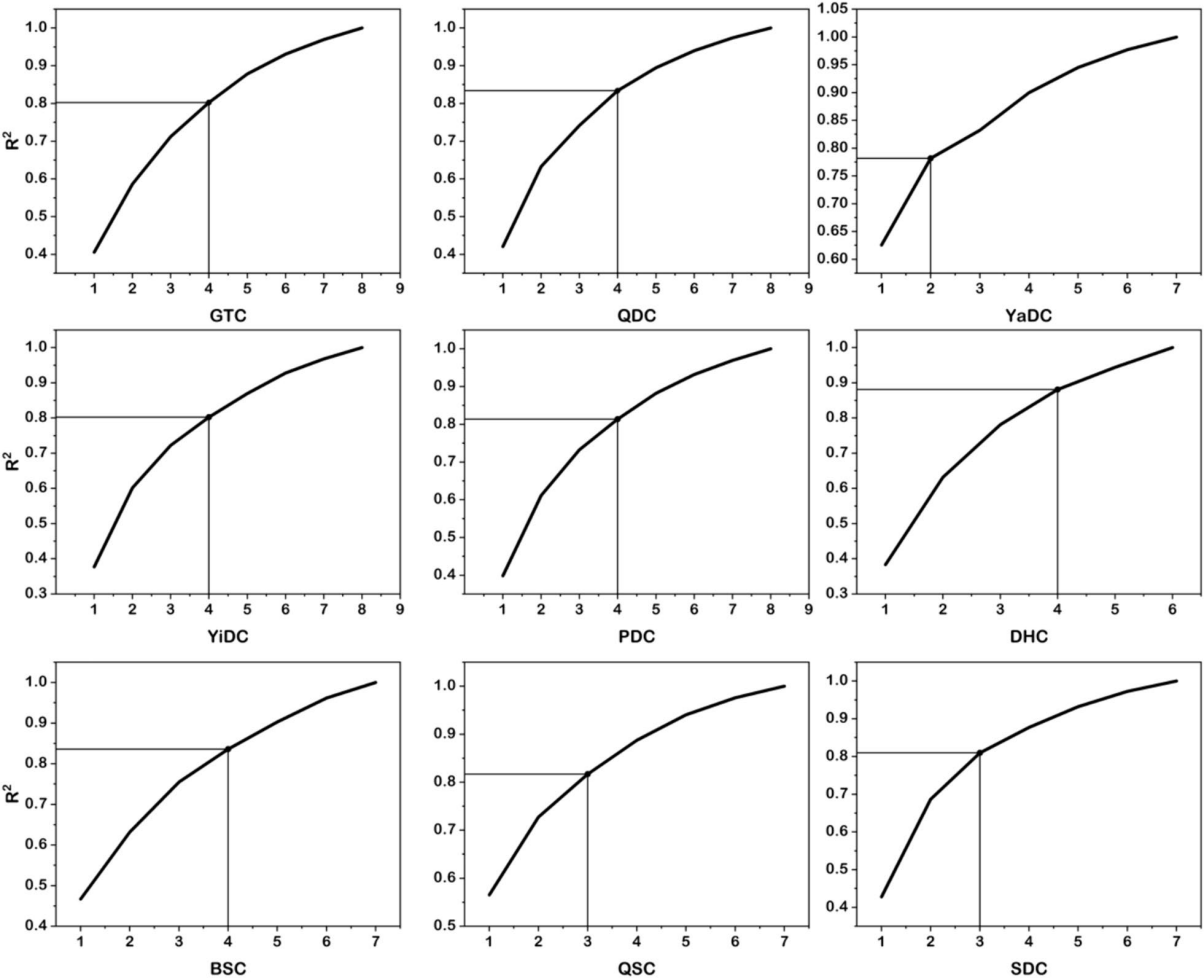
R^2^ for different item combinations. For YaDC, an elbow point was selected where the item combination maximized the improvement in the AUC. For GTC, QDC, YiDC, PDC, DHC, BSC, QSC and SDC, the curves were relatively smooth, so we selected the item combination with the fewest number of items when the AUC exceeded 0.8

Table 2 presents the optimal performance of the supervised machine learning item selection models with the BC score as the dependent variable. Figure 4 demonstrates the improvement in R^2^ performance for these optimal models.

**Table 2:** The optimal item combinations for the BC score as the dependent variable and their corresponding algorithms

	GTC	QDC	YaDC	YiDC	PDC	DHC	BSC	QSC	SDC
1 item	8	3	19	35	49	60	40	9	31
	DecisiontreeRegressor	DecisionTreeRegressor	RandomForestRegressor	XGBRegressor	GradientBoostingRegressor	RandomForestRegressor	ExtraTreesRegressor	GradientBoostingRegressor	XGBRegressor
	8, 21	3, 6	19, 52	20, 35	49, 50	39, 59	37, 40	9, 10	24, 31
	RandomForestRegressor	RandomForestRegressor	ExtraTreesRegressor	RandomForestRegressor	GradientBoostingRegressor	RandomForestRegressor	DecisionTreeRegressor	RandomForestRegressor	DecisionTreeRegressor
3 items	1, 8, 21	3, 6, 26	17, 19, 52	20, 35, 44	15, 49, 50	39, 56, 59	37, 40, 43	9, 10, 14	24, 30, 34
	DecisionTreeRegressor	ElasticNetCV	ExtraTreesRegressor	RidgeCV	RandomForestRegressor	RandomForestRegressor	ExtraTreesRegressor	ZeroCount	ElasticNetCV
	DecisionTreeRegressor	RandomForestRegressor		ExtraTreesRegressor				ExtraTreesRegressor	RandomForestRegressor
	DecisionTreeRegressor								
4 items	2, 8, 21, 53	3, 5, 6, 26	17, 18, 19, 52	20, 44, 46, 57	15, 28, 49, 50	39, 48, 56, 60	27, 37, 40, 43	9, 10, 14, 47	24, 30, 31, 34
	XGBRegressor	ExtraTreesRegressor	LassoLarsCV	XGBRegressor	RobustScaler	GradientBoostingRegressor	RidgeCV	RobustScaler	RidgeCV
		RobustScaler			RBFSampler		XGBRegressor	RBFSampler	XGBRegressor
		ElasticNetCV			RidgeCV			MinMaxScaler	
					ElasticNetCV			RidgeCV	
5 items	2, 8, 21, 53, 54	3, 5, 6, 22, 26	17, 18, 21, 22, 52	16, 38, 44, 46, 57	15, 28, 50, 51, 58	39, 41, 48, 56, 60	27, 37, 40, 43, 45	9, 10, 12, 14, 47	24, 25, 30, 31, 34
	ElasticNetCV	RobustScaler	MaxAbsScaler	RidgeCV	LinearSVR	RidgeCV	LinearSVR	RobustScaler	XGBRegressor
	RandomForestRegressor	RobustScaler	XGBRegressor	ElasticNetCV		PolynominalFeatures		LinearSVR	RidgeCV
		RidgeCV		RandomForestRegressor		RidgeCV			
6 items	2, 7, 21, 27, 53, 54	2, 3, 5, 6, 22, 26	17, 18, 21, 22, 52, 55	16, 35, 38, 44, 46, 57	13, 15, 28, 50, 51, 58	39, 41, 48, 56, 59, 60	27, 36, 37, 40, 43, 45	9, 10, 11, 12, 14, 47	23, 24, 25, 30, 31, 34
	MinMaxScaler	ElasticNetCV	AdaBoostRegressor	GradientBoostingRegressor	RidgeCV	LassoLarsCV	XGBRegressor	DecisionTreeRegressor	RobustScaler
	ZeroCount	GradientBoostingRegressor	RidgeCV				RidgeCV	StandardScaler	ElasticNetCV
	GradientBoostingRegressor							RidgeCV	RandomForestRegressor
7 items	1, 7, 8, 21, 27, 53, 54	2, 3, 5, 6, 7, 22, 26	17, 18, 19, 21, 22, 52, 55	16, 20, 29, 35, 44, 46, 57	13, 15, 28, 42, 50, 51, 58		27, 33, 36, 37, 40, 43, 45	8, 9, 10, 11, 12, 14, 47	23, 24, 25, 30, 31, 32, 34
	ElasticNetCV	RidgeCV	LassoLarsCV	LinearSVR	RobustScaler		FunctionUnion	LinearSVR	SGDRegressor
	RandomForestRegressor				ZeroCount		RidgeCV		StandardSScaler
					LassoLarsCV				RidgeCV
8 items	1, 2, 7, 8, 21, 27, 53, 54	2, 3, 4, 5, 6, 7, 22, 26		16, 20, 29, 35, 38, 44, 46, 57	13, 15, 28, 42, 49, 50, 51, 58				
	RidgeCV	LassoLarsCV		RidgeCV	SGDRegressor				

The specific meanings represented by the items are found in the supplementary materials

In Fig. 4, a clear elbow point is shown for the YaDC model with a two-item combination (item 19: cold aversion and item 52: cold sensitivity). Other BC score predictive models did not exhibit significant elbow points. Accordingly, the item combinations for the final BC scores prediction were selected from the models with an R^2^ exceeding 0.8 that used the fewest predictors. Specifically, the models for GTC, QDC, YiDC, PDC, DHC, and BSC included the first four items (GTC: item 2: tiredness, item 8: depression, item 21: cold intolerance, item 53: adaptability; QTC: item 3: breathlessness, item 5: dizziness, item 6: quietude, item 26: hyperhidrosis; YiDC: item 20: localized hotness, item 44: dry eyes, item 46: thirstiness, item 57: constipation; PDC: item 15: lethargy, item 28: oily T-zone, item 49: sticky mouth, item 50: flabby abdomen; DHC: item 39: oily skin, item 48: bitter mouth, item 56: sticky stools, item 60: wet scrotum/yellowing leukorrhea; BSC: item 27: forgetfulness, item 37: pain, item 40: hyperpigmentation, item 43: dark circles, respectively). Meanwhile, the QSC and SDC models incorporated the first three items (QSC: item 9: anxiety, item 10: vulnerability, item 14: sighing; SDC: item 24: chronic rhinitis, item 30: allergies, item 34: dermatographism, respectively), aligning with this criterion. For predicting BC scores, the models yielded R^2^ values ranging from 0.785 to 0.879 (shown in Table 3). According to general guidelines that R^2^ values of 0.75 and 0.50 are considered substantial and moderate, respectively, of predictive performance [20], all of these models had excellent predictive capacities. The range of RMSE values for these models was between 6.241 and 9.877, and the MAPE values ranged from 10.868 to 39.337.

**Table 3:** Evaluation of the automated machine learning results based on the appropriate supervised machine learning methods

Subscale	The BC classification as the target variable					The BC score as the target variable				
	Items	Algorithms	AUC	Accy	F1 score	Items	Algorithms	R^2^	RMAE	MAPE
GTC	4, 8, 21, 27	RobustScale XGBClassifier	0.891	0.878	0.417	2, 8, 21, 53	XGBRegressor	0.799	7.162	10.868
QDC	3, 6	XGBClassifier	0.888	0.823	0.741	3, 5, 6, 26	ExtraTreesRegressor RobustScaler ElasticNetCV	0.835	6.749	21.513
YaDC	19, 52	XGBClassifier	0.946	0.884	0.804	19, 52	ExtratreesRegressor	0.785	9.877	39.337
YiDC	20, 35	XGBClassifier	0.890	0.857	0.561	20, 44, 46, 57	XGBRegressor	0.804	6.740	27.560
PDC	49, 50	MLPClassifier	0.890	0.853	0.685	15, 28, 49, 50	RobustScaler RBFSampler RidgeCV ElasticNetCV	0.815	7.147	28.133
DHC	39, 59	ExtraTreesClassifier	0.894	0.835	0.693	39, 48, 56, 60	GradientboostingRegressor	0.879	6.241	22.208
BSC	40, 43	MLPClassifier	0.897	0.857	0.641	27, 37, 40, 43	RidgeCV XGBRegressor	0.836	6.720	21.582
QSC	9	GaussianNB	0.857	0.819	0.569	9, 10, 14	ZeroCount ExtraTreesRegressor	0.814	7.805	29.207
SDC	24, 31	GradientBoostingClassifier	0.942	0.936	0.603	24, 30, 34	ElasticNetCV RandomForestRegressor	0.811	6.575	34.079

The specific meanings represented by the items are found in the supplementary materials

### Item selection based on the unsupervised machine learning algorithm

**Fig. 5:**
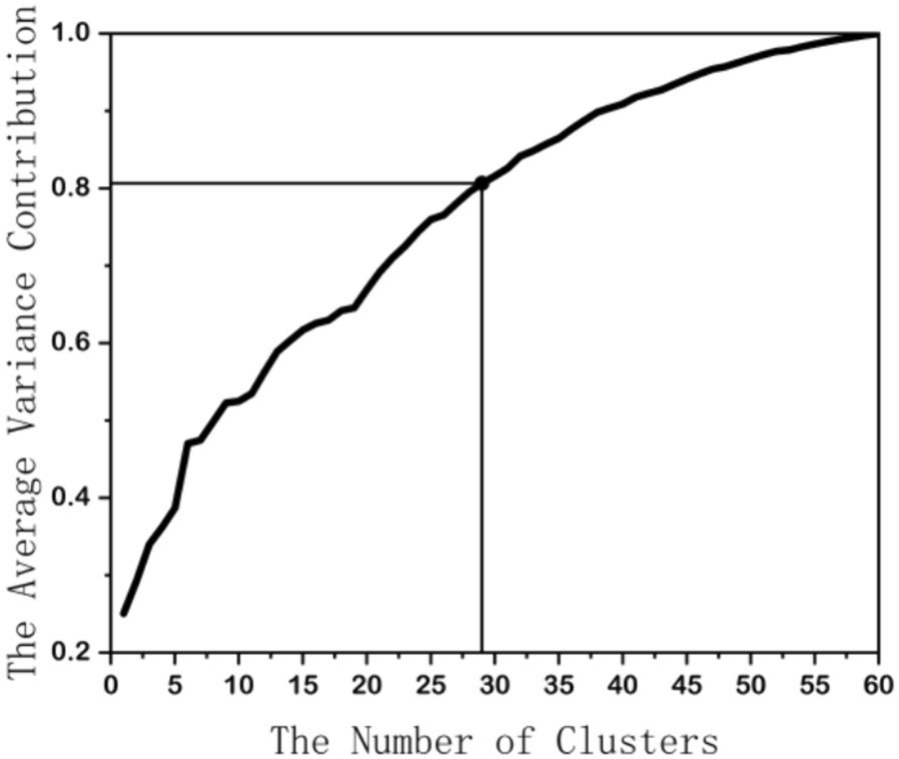
The average variance contribution of the representative items

For all possible numbers of clusters, the average variance contribution of all representative items within their respective clusters is illustrated in Fig. 5. When the number of clusters was set to 29, the representative items accounted for 80% of the variance within each cluster, on average. To further assess the predictive capability of these items for BC classification or score, the appropriate supervised machine learning models were constructed by TPOT, with these 29 items as independent variables and BC classifications or scores as dependent variables. The specific predictive algorithms and their corresponding performances are detailed in Table 4.

**Table 4:** Evaluation of varclus results based on the appropriate supervised machine learning methods

Subscale	Items	The BC classification as the target variable				The BC score as the target variable			
		Algorithms	AUC	Accy	F1 score	Algorithms	R^2^	RMAE	MAPE
GTC	2, 27, 53	XGBClassifier	0.847	0.865	0.294	ZeroCount RandomForestRegressor	0.676	9.143	14.470
QDC	2, 4, 6, 22, 26	RobustScaler MLPClassifier MLPClassifier	0.965	0.899	0.858	PolynomialFeatures LassoLarsCV	0.888	5.602	17.021
YaDC	19, 22, 52, 55	MaxAbsScaler RobustScaler MLPClassifier	0.960	0.904	0.841	GradientBoostingRegressor RidgeCV	0.866	8.608	35.056
YiDC	16, 20, 29, 44, 57	MLPClassifier	0.960	0.909	0.754	PolynomialFeatures LassoLarsCV	0.817	6.593	29.433
PDC	50, 51, 58	MLPClassifier	0.924	0.867	0.719	RandomForestRegressor	0.694	9.188	38.755
DHC	39, 48, 60	ExtraTreesClassifier	0.934	0.874	0.765	RobustScaler RBFSampler LassoLarsCV	0.769	8.616	31.898
BSC	27, 33, 36, 37, 43	XGBClassifier MLPClassifier	0.951	0.902	0.773	PolynomialFeatures RidgeCV	0.843	6.567	23.705
QSC	10	MLPClassifier	0.855	0.820	0.611	AdaBoostRegressor	0.549	12.135	48.239
SDC	23, 25, 31	ZeroCount MLPClassifier	0.963	0.950	0.672	XGBRegressor	0.777	7.206	42.381

The specific meanings represented by the items are found in the supplementary materials

In terms of predicting BC classifications, the selected items achieved AUC values ranging from 0.847 to 0.965, prediction accuracies ranging from 0.820 to 0.950 and F1 scores from 0.294 to 0.858. For predicting BC scores, the models yielded R^2^ values from 0.549 to 0.888, RMSE values from 5.602 to 12.135 and MAPE values from 14.470 to 48.239 (shown in Table 4). According to the criteria mentioned above [20, 21], most models exhibited outstanding predictive performance, while a few were considered to have moderate predictive capability.

### Comparison between automated machine learning and unsupervised machine learning

**Fig. 6:**
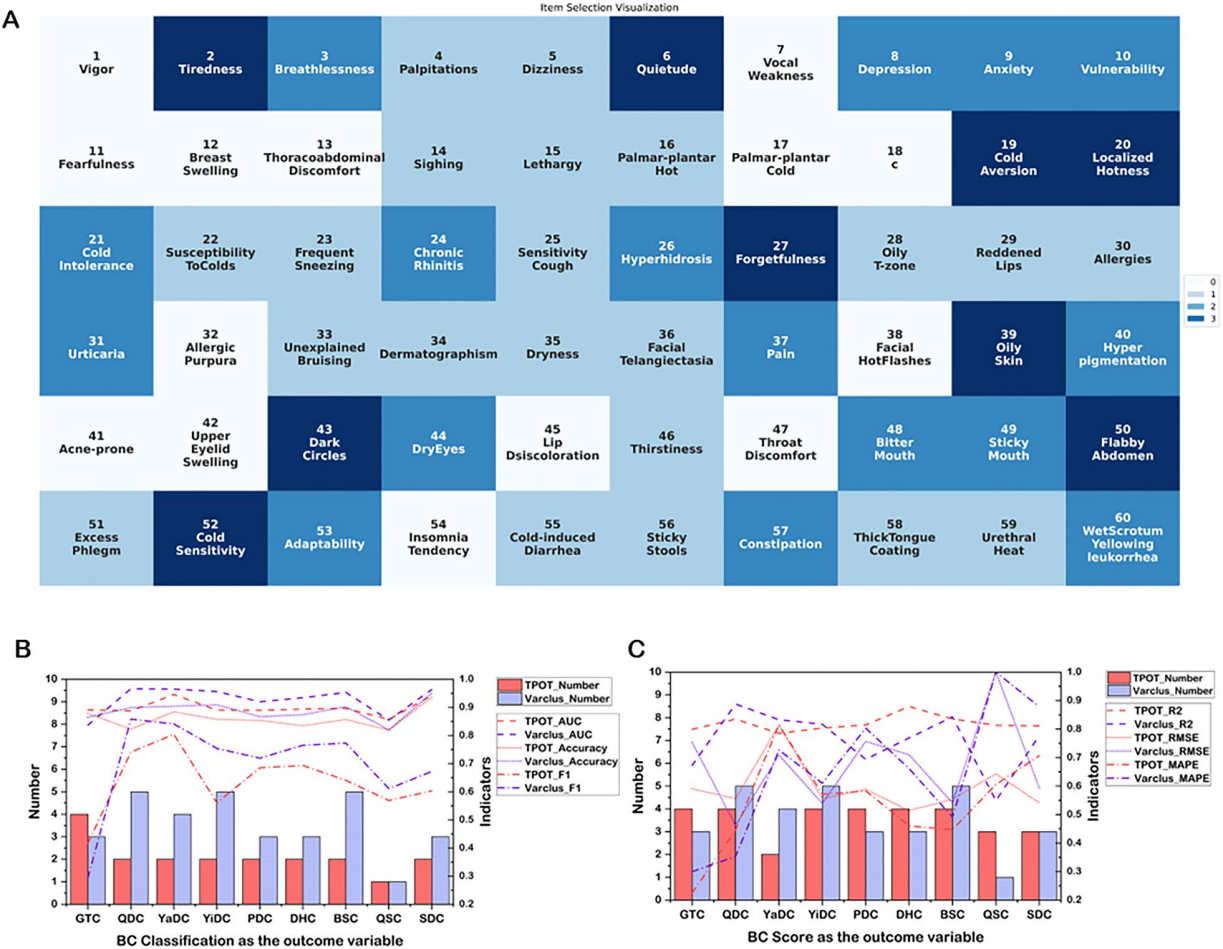
Comparison of prediction performance using the appropriate supervised machine learning for items selected based on TPOT and varclus. **A** The frequency of items selected based on TPOT and varclus. **B** Performance of items selected based on TPOT and varclus in predicting BC classifications using the appropriate supervised machine learning method. **C** Performance of items selected based on TPOT and varclus in predicting BC scores using the appropriate supervised machine learning method. In **C**, the RMSE measure is represented as RMSE/RMSEmax, and the MAPE is represented as MAPE/MAPEmax

We conducted three types of item selection procedures from the CCMQ: items selected by automated machine learning algorithms with classifications as the target variables, items selected by automated machine learning algorithms with scores as the outcome variables, and representative items selected by unsupervised learning. Figure 6A illustrates the frequency of items selected based on the three types of item selection procedures. As shown in Fig. 6A, item 2 (i.e., tiredness), item 6 (i.e., quietude), item 19 (i.e., cold aversion), item 20 (i.e., localized hotness), item 27 (i.e., forgetfulness), item 39 (i.e., oily skin), item 43 (i.e., dark circles), item 50 (i.e., flabby abdomen), and item 52 (i.e., cold sensitivity) were consistently chosen by these three types of item selection procedures, indicating that they contain multiple pieces of information, including associations with BC classifications and scores, as well as relationships with other items. Additionally, the supervised machine learning algorithms consistently selected item 3 (i.e., breathlessness), item 8 (i.e., depression), item 9 (i.e., anxiety), item 21 (i.e., cold intolerance), item 24 (i.e., chronic rhinitis), item 40 (i.e., hyperpigmentation), and item 49 (i.e., ticky mouth) for predicting BC classifications and scores. Different from the items selected by the supervised machine learning algorithms, the varclus algorithm selected item 16 (i.e., palmar-plantar hot), item 22 (i.e., susceptibility to colds), item 23 (i.e., frequent sneezing), item 25 (i.e., sensitivity cough), item 29 (i.e., reddened lips), item 33 (i.e., unexplained bruising), item 36 (i.e., facial telangiectasia), item 51 (i.e., excess phlegm), item 55 (i.e., cold-induced diarrhea), and item 58 (i.e., thick tongue coating) to predict the BC classifications or scores.

In the prediction of classifications, the items selected based on TPOT and those selected by varclus, using the appropriate prediction models, demonstrated similar AUCs, accuracies and F1 scores (Fig. 6B). However, except for the GTC model, TPOT selected fewer items for the biased BC classifications compared to varclus.

In the prediction of scores for BC, such as GTC, PDC, DHC, QSC, and SDC, the items selected based on TPOT demonstrated notable advantages in comparison with those selected by varclus (Fig. 6C). However, in the prediction of scores for BC types such as QDC and YaDC, selections made by varclus and its corresponding predictive algorithms exhibited superior performance. For the YiDC and BSC score predictions, the performances of the items chosen by TPOT and varclus were similar. Overall, while the number of items selected by TPOT and varclus remains similar, the selections made by TPOT yield a higher average R^2^ and lower average RMSE and MAPE.

## Discussion

Self-report questionnaires are recognized in clinical practice as effective tools for quantifying abstract concepts, aiding in assessments of disease risk [23–25]. As the use of questionnaires grows, there is an increased demand for them to possess strong predictive capabilities and to facilitate rapid disease determination. In this study, we utilized machine learning techniques for rapidly determining BCs.

The comparative analysis between AutoML and unsupervised machine learning in terms of item selection for BC classifications and scores revealed a slight advantage for AutoML. The reason might be that the predictive targets were predefined dimensions of the CCMQ. Thus, AutoML are recommended for predicting original BC classifications or scores.

There was consistency among the different item selection procedures. Items 2 (i.e., tiredness), 6 (i.e., quietude), 19 (i.e., cold aversion), 20 (i.e., localized hotness), 27 (i.e., forgetfulness), 39 (i.e., oily skin), 43 (i.e., dark circles), 50 (i.e., flabby abdomen), and 52 (i.e., cold sensitivity) were selected by both supervised machine learning algorithms and varclus. These items were consistently selected because they may encompass information in other items within a given BC type. Given that constitutions are closely related to the development of certain diseases [26–29] special attention may need to be given to these factors for rapid disease prediction. For example, items 2 (i.e., tiredness), 27 (i.e., forgetfulness), 19 (i.e., cold aversion), 52 (i.e., cold sensitivity), and 20 (i.e., localized hotness), which are related to the QDC, YaDC and YiDC, can be core predictors of chronic fatigue syndrome (CFS), generalized anxiety disorder, depression, and anemia [26, 30–32]. Items 39 (i.e., oily skin) and 50 (i.e., flabby abdomen) are indicators of DHC and PDC, respectively, and can be used to predict the occurrence of metabolic diseases and polycystic ovary syndrome [33–35].

Previous research simplified scales by calculating feature importance using supervised machine learning algorithms for predicting total scores with item scores and retaining items with high feature importance [16, 17, 36]. However, this method faces the issue that different machine learning algorithms may assign items different weights [37]. We may select the appropriate supervised machine learning algorithms before calculating feature importance, but this chosen algorithm may not perform best when using the selected items to predict the original scores. In this study, we innovatively selected the best-performing model from those built using all possible combinations of items as input variables. Thus, the absolute improvement in the models’ predictive performance as the number of items used to predict the original BC classifications or scores increases can be calculated. For each possible number of items, the top three ranked item combinations in terms of predictive effectiveness, along with their corresponding algorithms, are listed in the supplemental material (Figure S1 and S2). Consequently, we can comprehensively understand the predictive capabilities of all possible item combinations and their corresponding algorithms and assist practitioners in selecting item combinations based on their understanding of TCM theories and specific needs in scale development (e.g., required reliability and validity, test efficiency).

In summary, this study has significant implications for the BC identification in clinical practice. Firstly, the machine learning algorithms proposed in this study enable rapid BC identification based on a subset of items. Secondly, by comparing different supervised and unsupervised machine learning algorithms, it is possible to gain deeper insights into how different items contribute to the various BC dimensions, thereby assisting clinical practitioners in achieving a more thorough understanding of these dimensions.

A major limitation of this study is the computational cost arising from the complexity of the algorithm combined with the large volume of data. Therefore, future research should focus on optimizing algorithms to enhance their processing speed and efficiency in big data environments. Also, in the future, we can further optimize the rapid body constitution identification process by integrating multimodal data, such as tongue and pulse diagnostics.

## Conclusion

The items in the CCMQ were shown to have varying information weights. The use of highly important items may assist in the rapid determination of BCs. The use of supervised machine learning algorithms with all the possible item combinations for predicting BC classifications or scores achieved acceptable and stable predictive performance. The top item combinations obtained by supervised machine learning algorithms for predicting BC classifications or scores were identified so that other researchers can make selections according to their needs.

## Supplementary Information

Supplementary Material 1

## Data Availability

All data included in this article are available from the Prof Qi Wang upon request.

## Abbreviations

CCMQ: Constitution of Chinese Medicine Questionnaire
BC: Body constitution
TCM: Traditional Chinese Medicine
AutoML: Automated machine learning
TPOT: Tree-based pipeline optimization tool
varclus: Variable clustering
GTC: Gentleness constitution
QDC: Qi-deficiency constitution
YaDC: Yang-deficiency constitution
YiDC: Yin-deficiency constitution
PDC: Phlegm-dampness constitution
DHC: Damp-heat constitution
BSC: Blood-stasis constitution
QSC: Qi-stagnation constitution
SDC: Special-diathesis constitution
AUC: Area under the curve
R^2^: R-squared
RMSE: Root mean square error.
MAPE: Mean absolute percentage error
