# Supplementary material for: Machine learning-assisted rapid determination for traditional Chinese Medicine Constitution
Source: Chin Med. 2024 Sep 15;19:127. doi: 10.1186/s13020-024-00992-0 (PMC11403957; doi:10.1186/s13020-024-00992-0)
Supplement: Supplementary file 1 — Supplementary Material 1 [file 13020_2024_992_MOESM1_ESM.docx]

Supplementary Materials

Table

Table S1 Title and abbreviation corresponding to the item

| Item | Abbreviation | Scale |
| --- | --- | --- |
| (1) Were you energetic? | Vigor | GTC |
| (2) Did you get tired easily? | Tiredness | GTC, QDC |
| (3) Did you experience shortness of breath? | Breathlessness | QDC |
| (4) Did you get palpitations? | Palpitations | QDC |
| (5) Did you get dizzy easily or become dizzy when standing up? | Dizziness | QDC |
| (6) Did you prefer quietness and not like to talk? | Quietude | QDC |
| (7) Was your voice weak when talking? | Vocal Weakness | GTC, QDC |
| (8) Did you feel in low spirits and depressed? | Depression | GTC |
| (9) Did you easily feel anxious and worried? | Anxiety | QSC |
| (10) Did you feel overly sensitive, vulnerable or emotionally upset? | Vulnerability | QSC |
| (11) Were you easily scared or frightened? | Fearfulness | QSC |
| (12) Did you experience distention in the underarm or breast? | Breast Swelling | QSC |
| (13) Did you feel chest or abdominal stuffiness? | Thoracoabdominal Discomfort | PDC |
| (14) Did you sigh without reason? | Sighing | QSC |
| (15) Did your body feel heavy or lethargic? | Lethargy | PDC |
| (16) Did the palms of your hands or soles of your feet feel hot? | Palmar-plantar Hot | YiDC |
| (17) Did your hands or feet feel cold or clammy? | Palmar-plantar Cold | YaDC |
| (18) Did you feel cold easily in your abdomen, back, lower back or knees? | Localized Cold Aversion | YaDC |
| (19) Were you sensitive to cold and tended to wear more clothes than others? | Cold Aversion | YaDC |
| (20) Did your body and face feel hot? | Localized Hotness | YiDC |
| (21) Did you feel more vulnerable to the cold than others (winter coldness, air conditioners, fans, etc.)? | Cold Intolerance | GTC, YaDC |
| (22) Did you catch colds more easily than others? | Susceptibility To Colds | QDC, YaDC |
| (23) Did you sneeze even when you did not have a cold? | Frequent Sneezing | SDC |
| (24) Did you have a runny or stuffy nose even when you did not have a cold? | Chronic Rhinitis | SDC |
| (25) Did you cough due to seasonal changes, temperature changes or unpleasant odors? | Sensitivity Cough | SDC |
| (26) Did you sweat easily when your physical activity increased slightly? | Hyperhidrosis | QDC |
| (27) Did you forget things easily? | Forgetfulness | GTC, BSC |
| (28) Did you have an excessively oily forehead and/or T-zone? | Oily T-zone | PDC |
| (29) Were your lips redder than in the past? | Reddened Lips | YiDC |
| (30) Did you have allergies? (E.g. medicine, food, odors, pollen, pet dander or during seasonal or weather change etc.) | Allergies | SDC |
| (31) Did you get hives/urticaria easily? | Urticaria | SDC |
| (32) Did your skin have purpura (purple spots, ecchymosis) due to allergies? | Allergic Purpura | SDC |
| (33) Did black or purple bruises appear on your skin for no reason? | Unexplained Bruising | BSC |
| (34) Did your skin turn red and show traces when you scratched it? | Dermatographism | SDC |
| (35) Did your skin or lips feel dry? | Dryness | YiDC |
| (36) Did you have visible capillary (thread) veins on your cheeks? | Facial Telangiectasia | BSC |
| (37) Did you feel pain somewhere in your body? | Pain | BSC |
| (38) Did you experience hot flashes? | Facial Hot Flashes | YiDC |
| (39) Did your nose or your face feel greasy, oily, or shiny? | Oily Skin | DHC |
| (40) Did you have a dark face or get brown spots easily? | Hyperpigmentation | BSC |
| (41) Did you get acne or sores easily? | Acne-prone | DHC |
| (42) Did you have upper eyelid swelling? | Upper Eyelid Swelling | PDC |
| (43) Did you get dark circles under the eyes easily? | Dark Circles | BSC |
| (44) Did your eyes feel dry and you used eye drops? | Dry Eyes | YiDC |
| (45) Were your lips darker, more blue or purple than usual? | Lip Dsiscoloration | BSC |
| (46) Did you often feel parched and need to drink water? | Thirstiness | YiDC |
| (47) Did your throat feel strange (i.e., as if something was stuck or there was a lump in your throat)? | Throat Discomfort | QSC |
| (48) Did you have a bitter or strange taste in your mouth? | Bitter Mouth | DHC |
| (49) Did your mouth feel sticky? | Sticky Mouth | PDC |
| (50) Was your abdomen flabby? | Flabby Abdomen | PDC |
| (51) Did you have an abundance of phlegm, especially in your throat? | Excess Phlegm | PDC |
| (52) Did you feel uncomfortable when you drank or ate something cold, or did you avoid to drinking or eating cold items? | Cold Sensitivity | YaDC |
| (53) Could you adapt yourself to external natural or social environment changes? | Adaptability | GTC |
| (54) Did you easily experience insomnia? | Insomnia Tendency | GTC |
| (55) Did you easily contract diarrhea when you were exposed to cold or ate (or drank) something cold? | Cold-induced Diarrhea | YaDC |
| (56) Did you pass sticky stools and/or feel that your bowel movement was incomplete? | Sticky Stools | DHC |
| (57) Did you get constipated easily or have dry stools? | Constipation | YiDC |
| (58) Did your tongue have a thick coating? | ThickTongue Coating | PDC |
| (59) Did your urethral canal feel hot when you urinated, or did your urine have a dark color? | Urethral Heat | DHC |
| (60) Was your vaginal discharge yellowish (only for female interviewees)? /Was your scrotum always wet (only for male interviewees)? | WetScrotum  Yellowing leukorrhea | DHC |

Figure


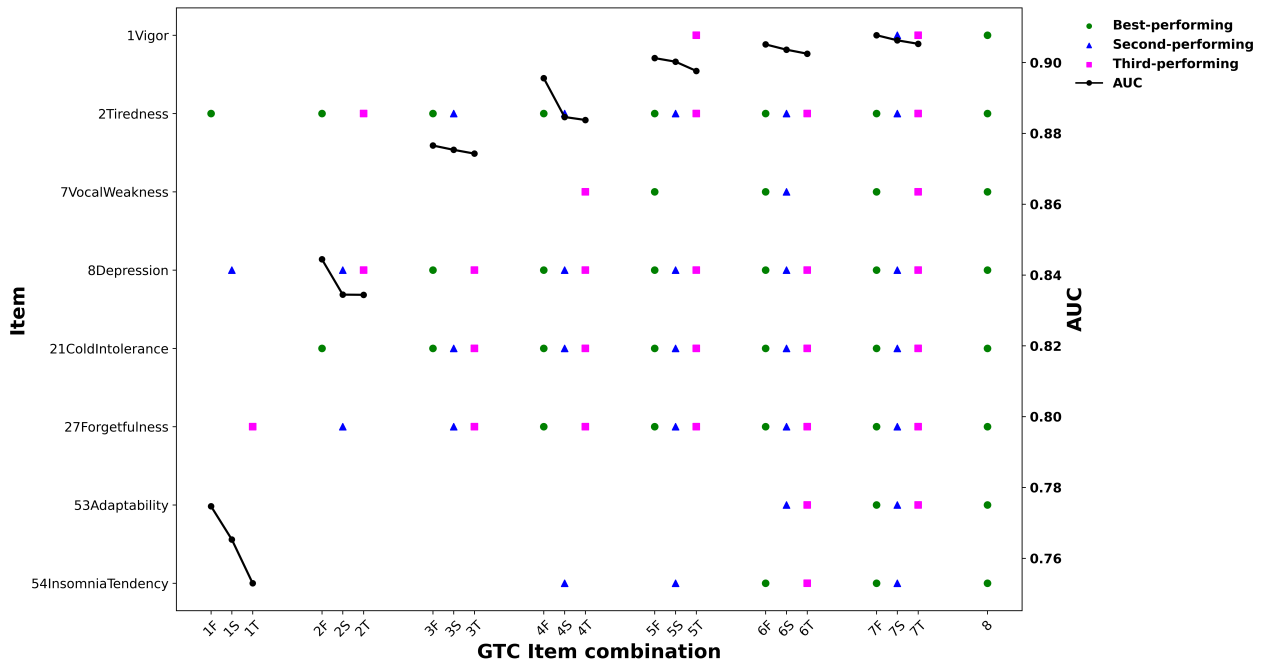


(A)


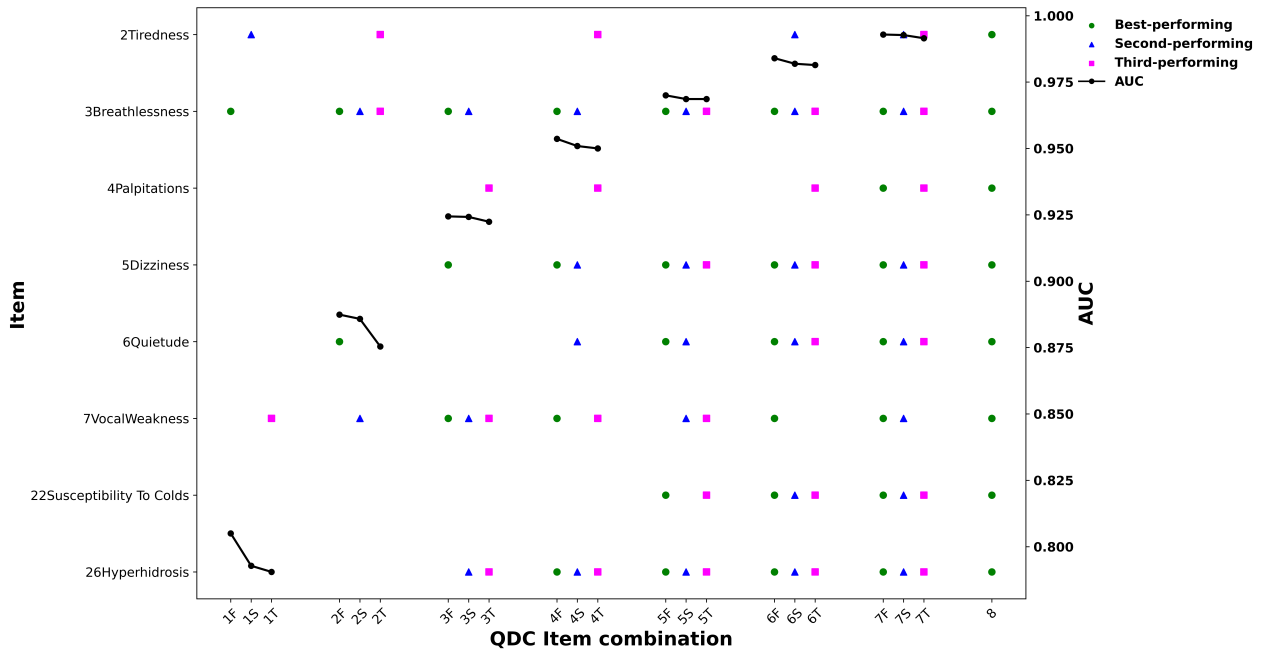


(B)


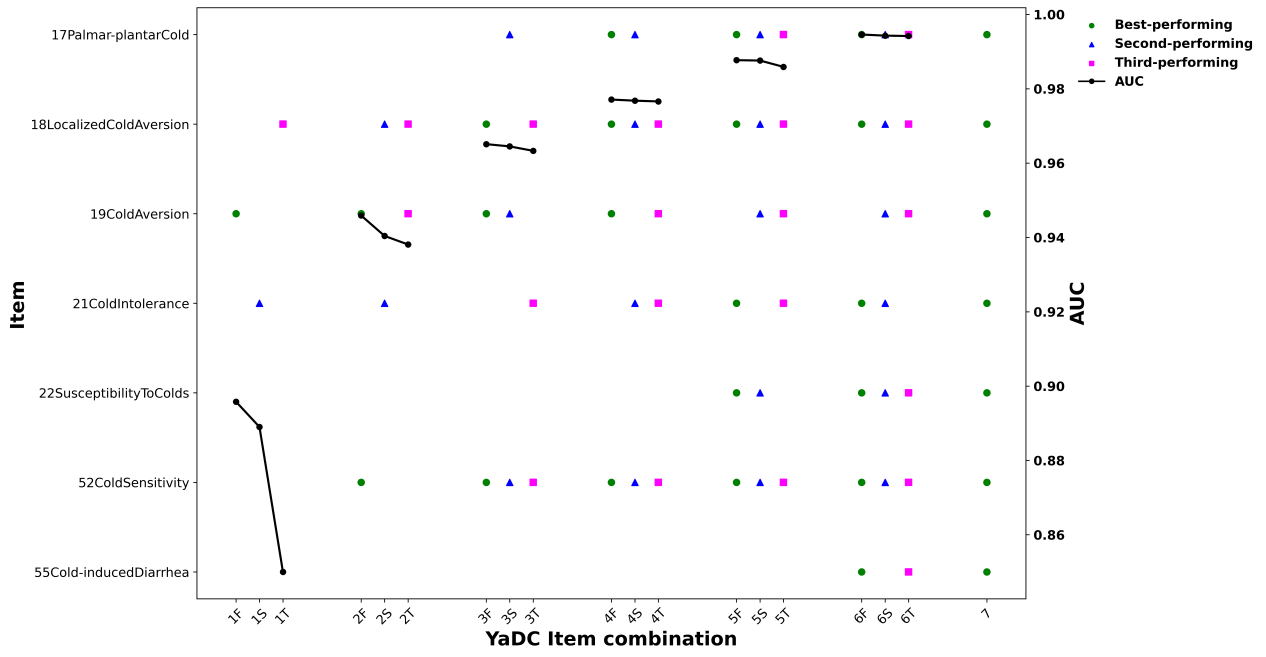


(C)


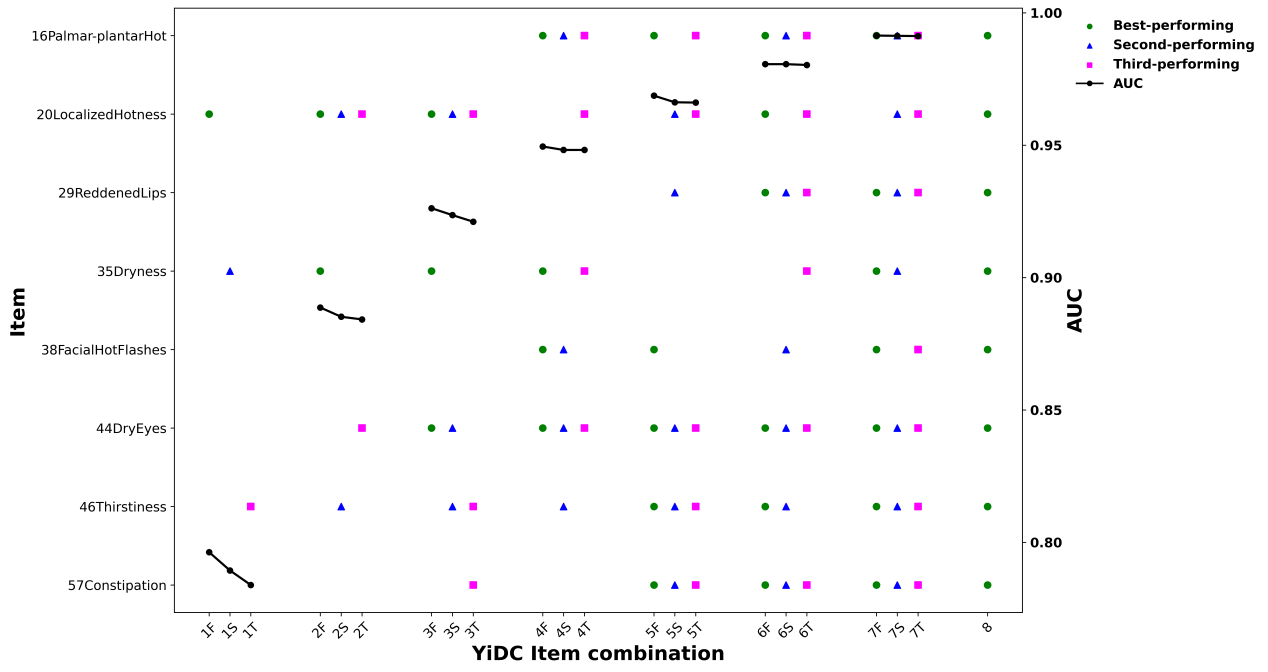


(D)


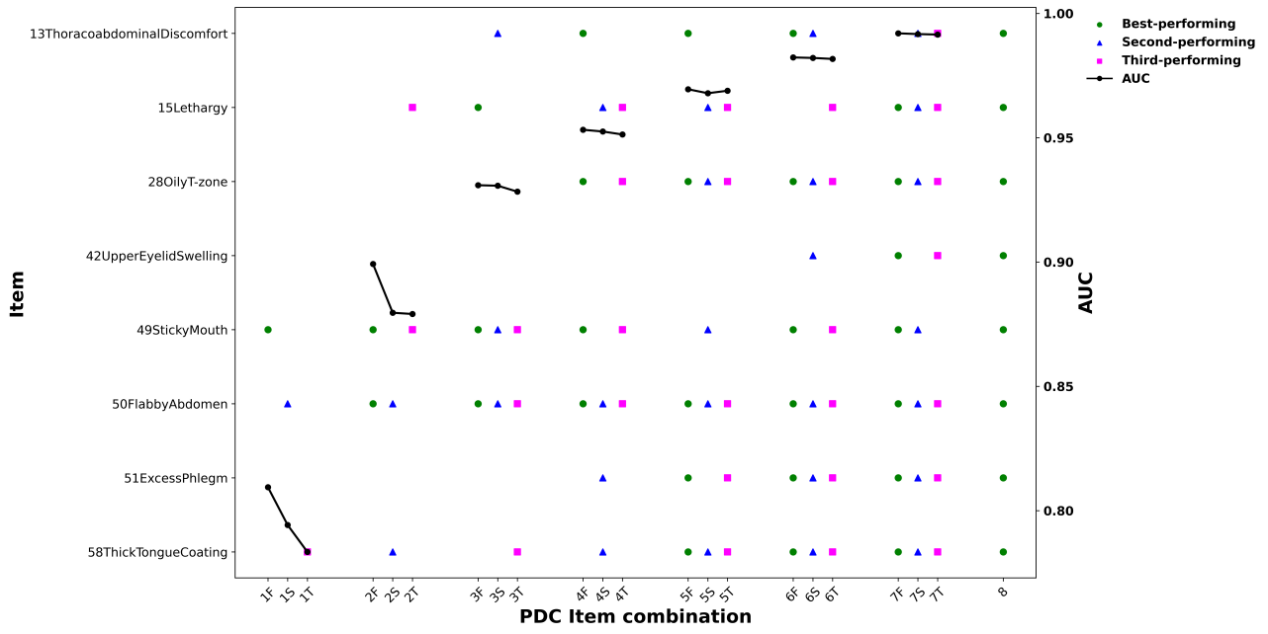


(E)


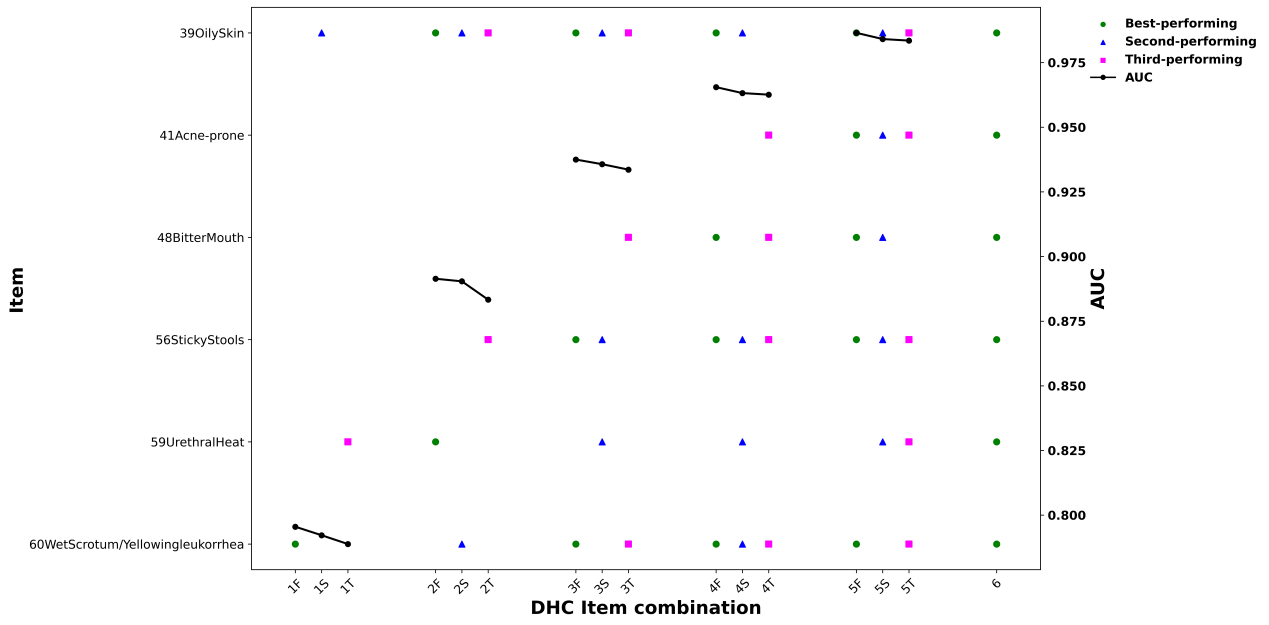


(F)


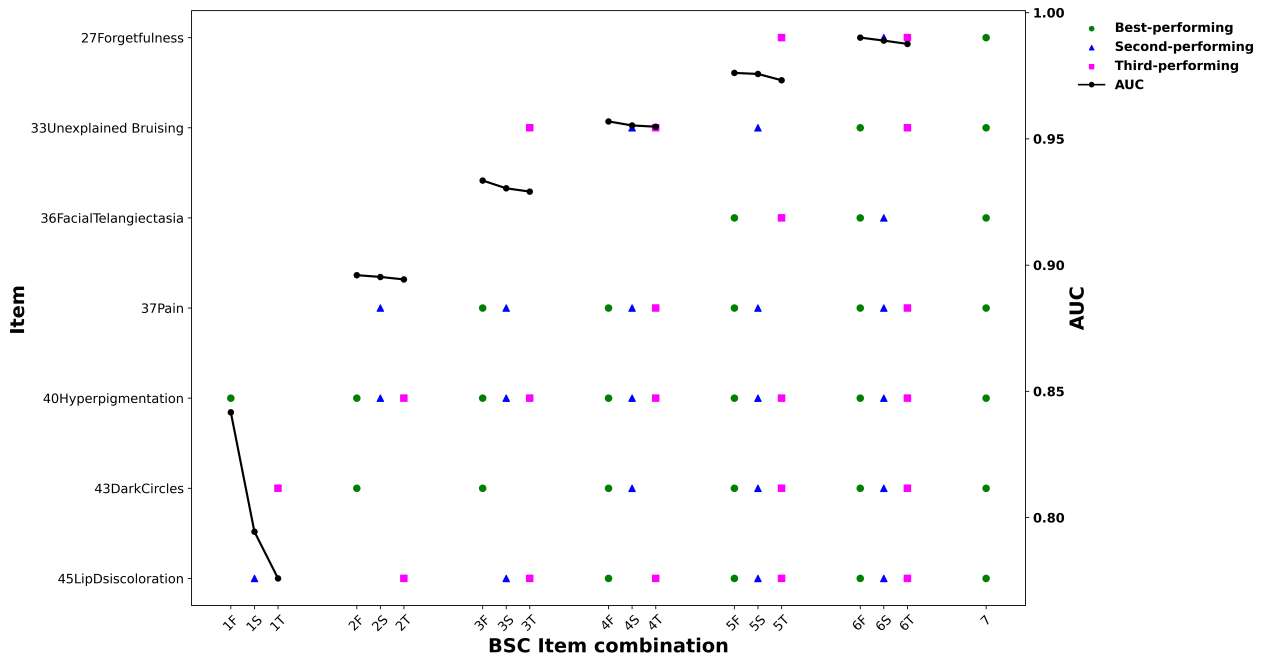


(G)


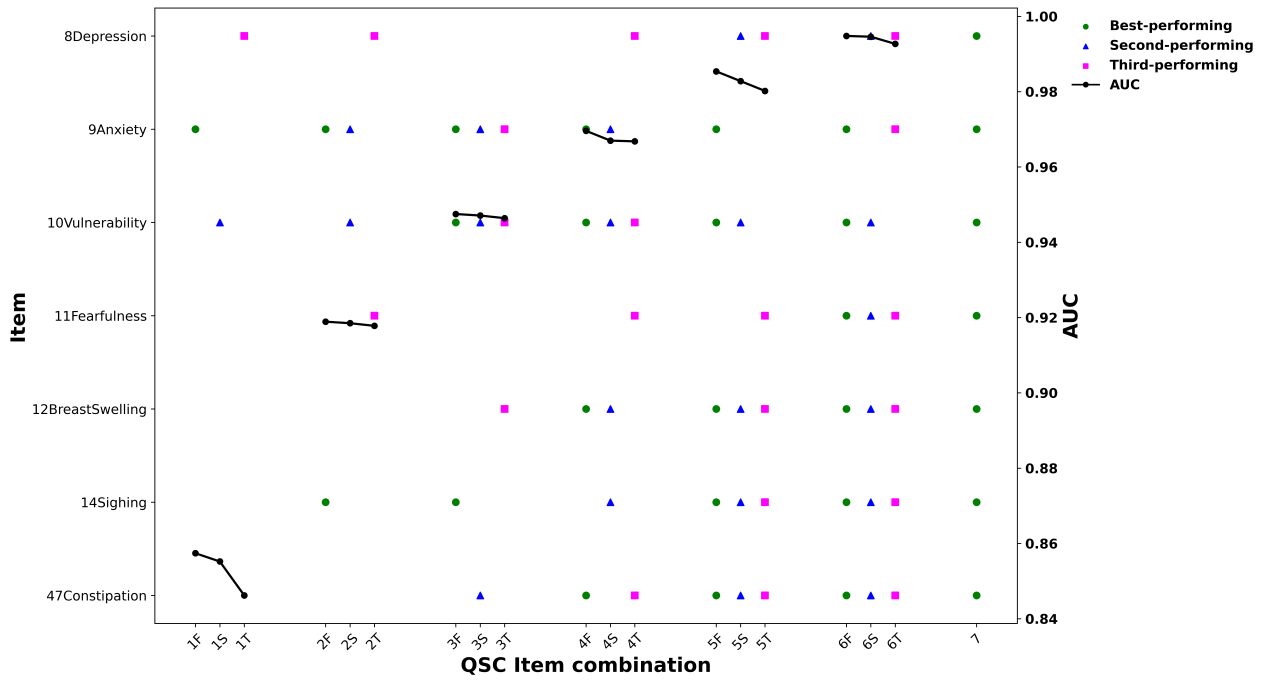


(H)


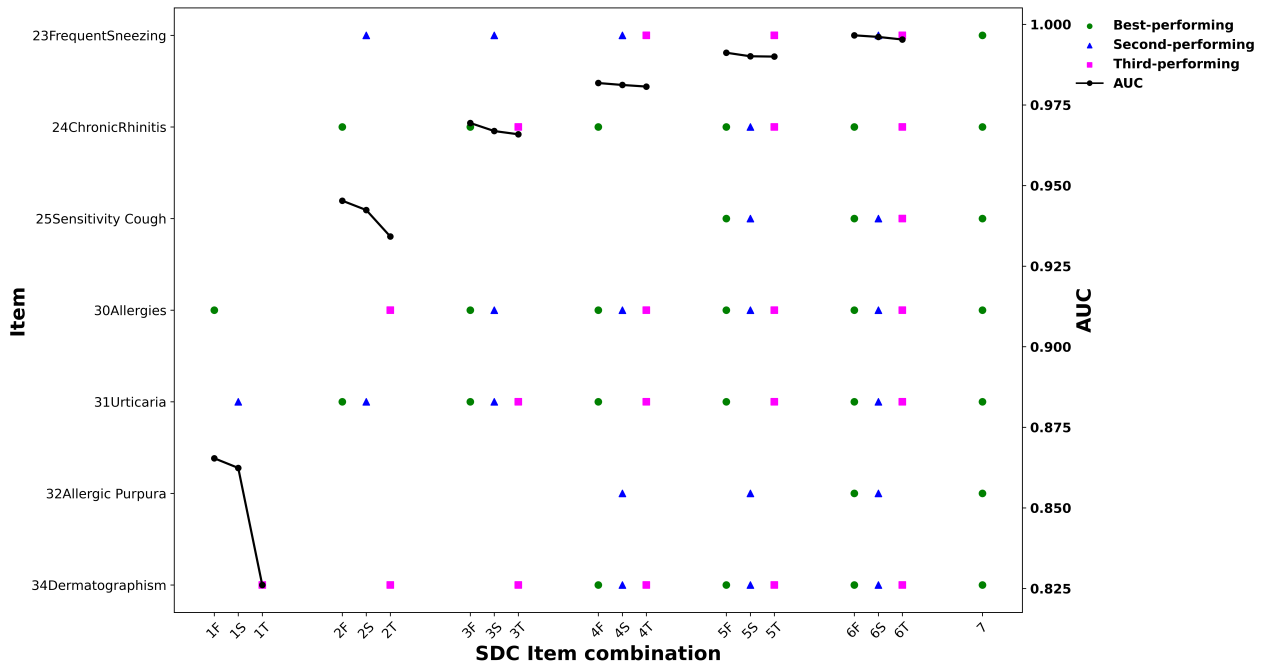


(I)

Figure S1 Top three item combinations and their corresponding AUC based on classification.

Note. The left vertical axis represents the specific item numbers and content included in the subscales of a particular body constitution, while the right vertical axis indicates the AUC. The horizontal axis represents different combinations of items, with F denoting the best-performing combination, S the second best, and T the third best. The positions of scatter points with specific shapes and colors illustrate which items are included in item combinations of varying performance and item numbers, while the right vertical axis shows the AUC for these item combinations. A, B, C, D, E, F, G, H, and I represent the results for GTC, QDC, YaDC, YiDC, PDC, DHC, BSC, QSC, and SDC, respectively.


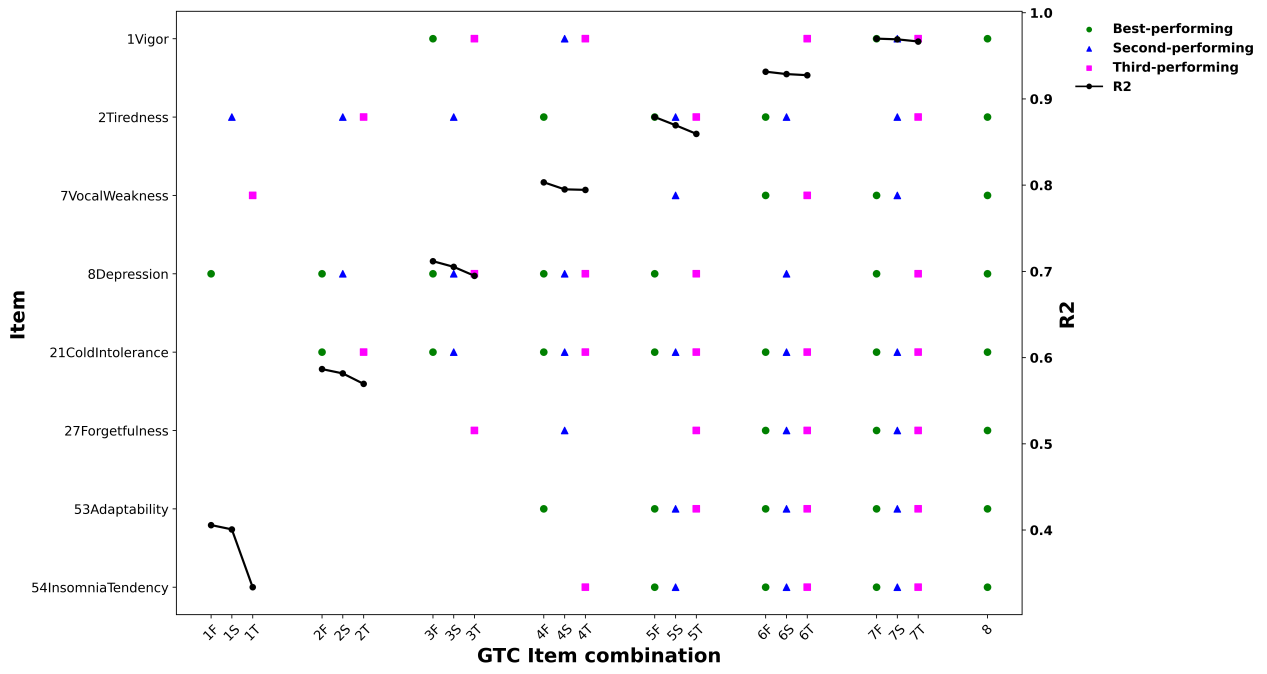


(A)


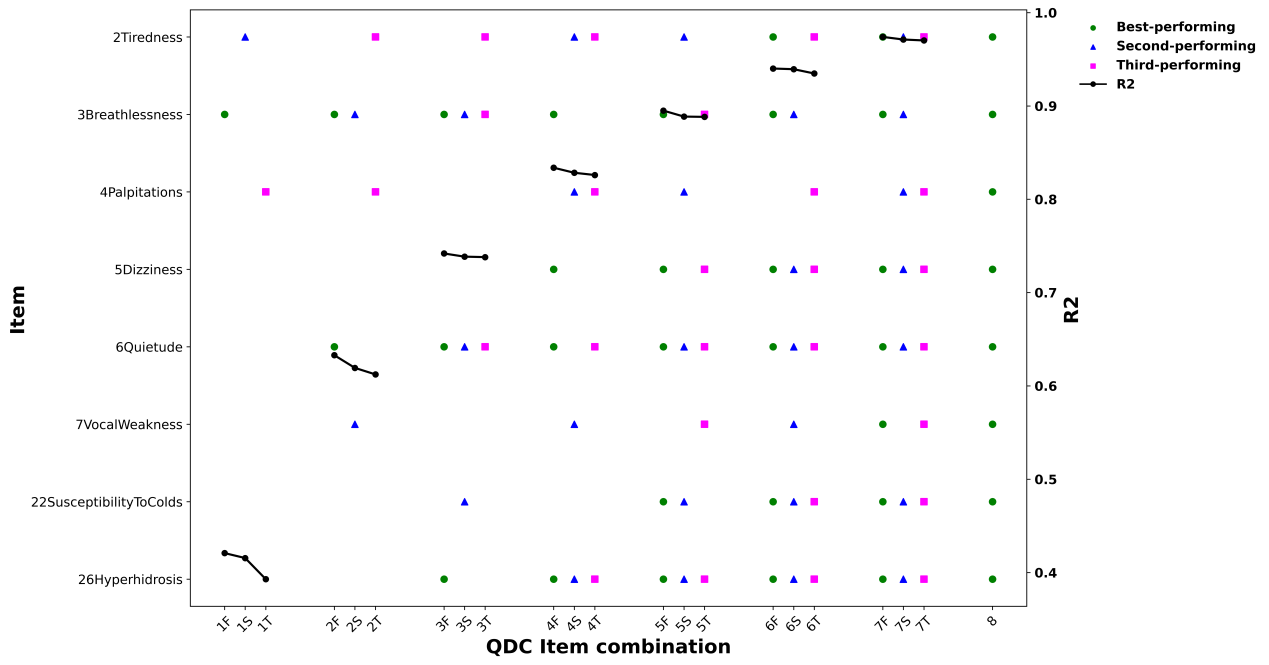


(B)


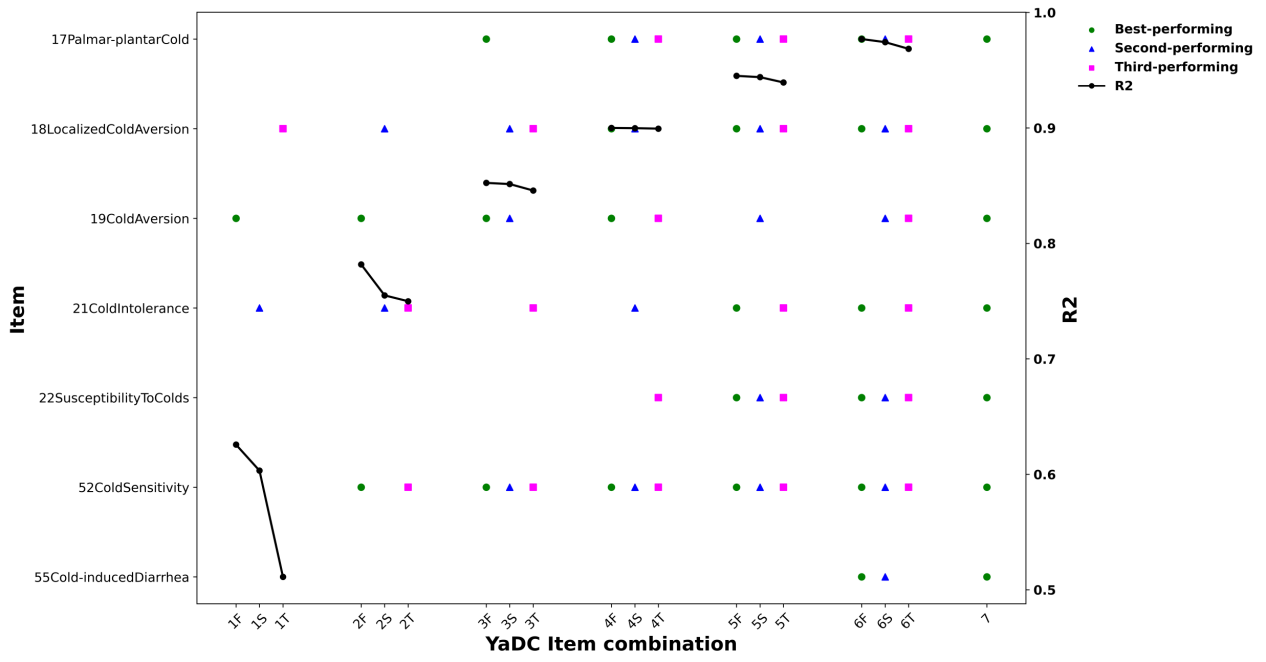


(C)


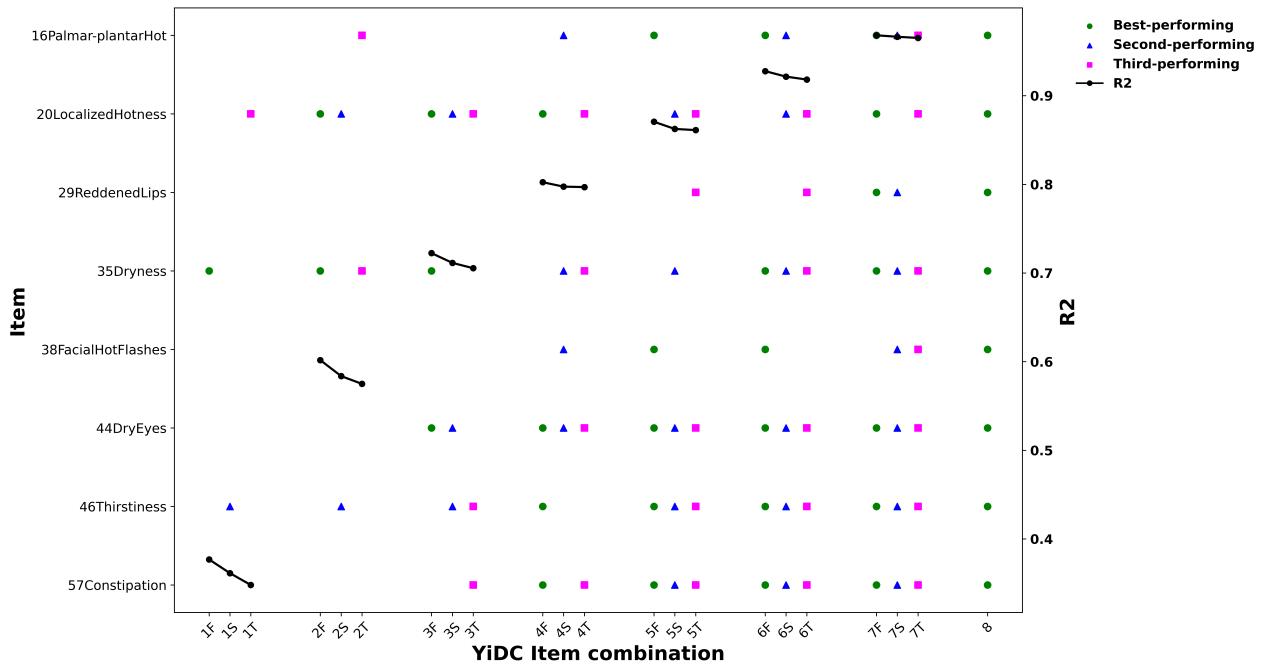


(D)


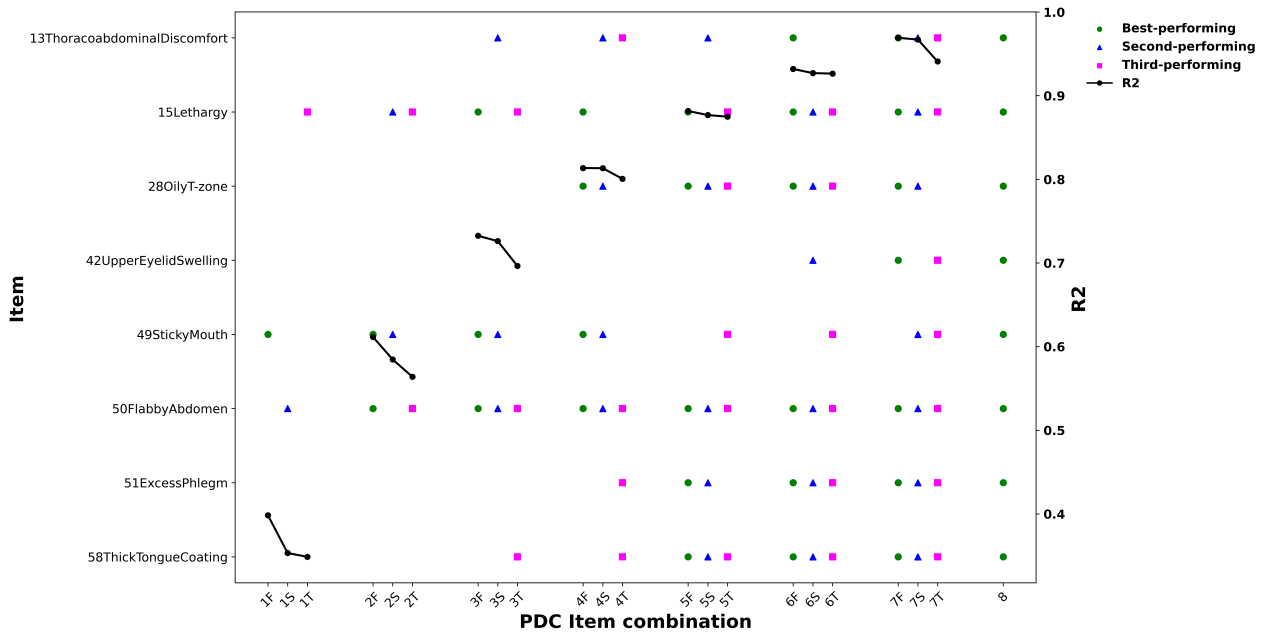


(E)


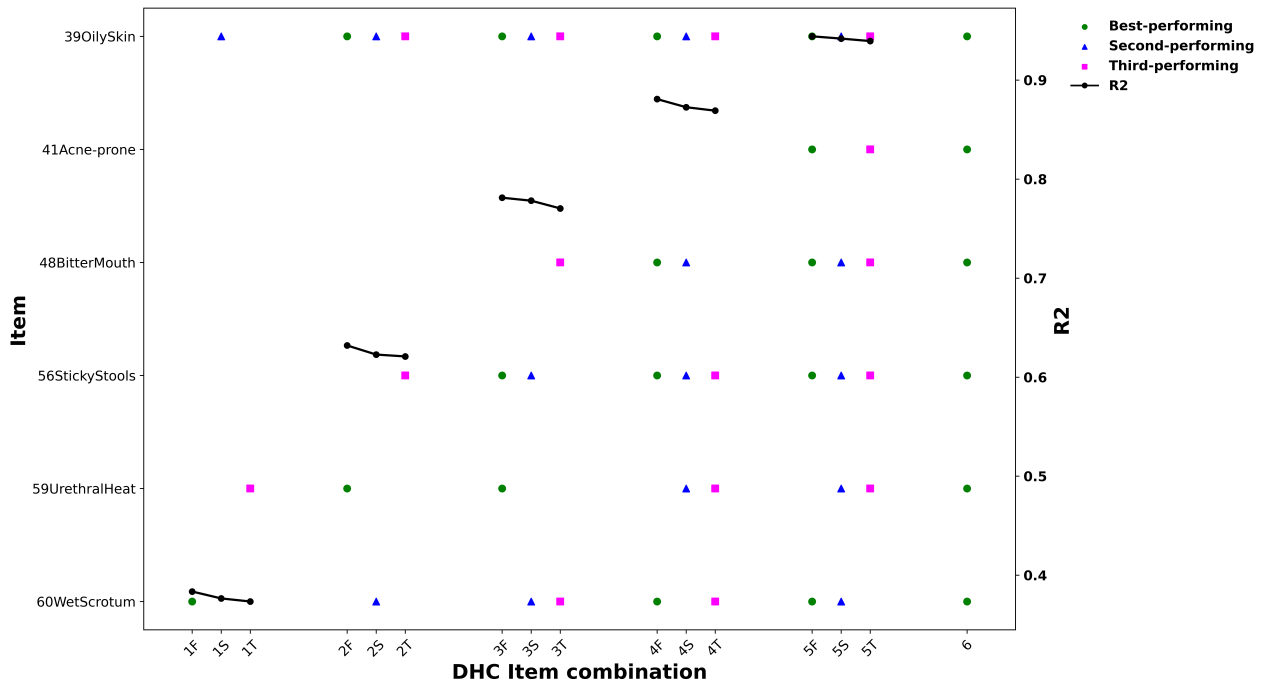


(F)


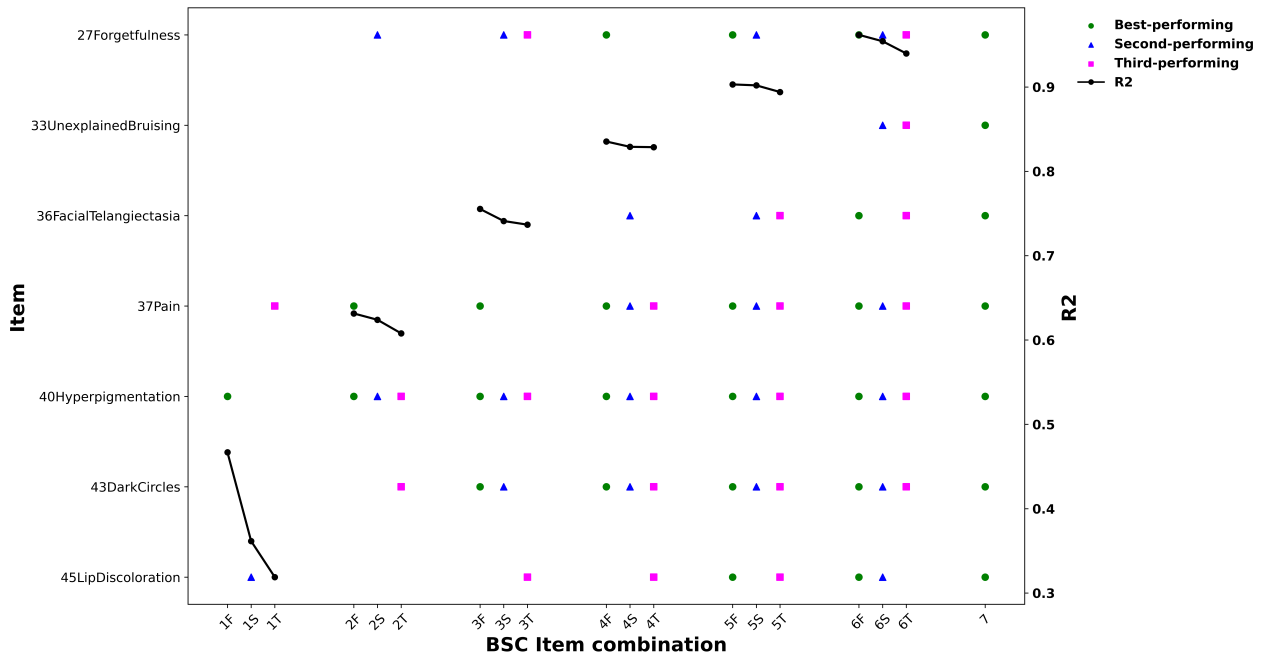


(G)


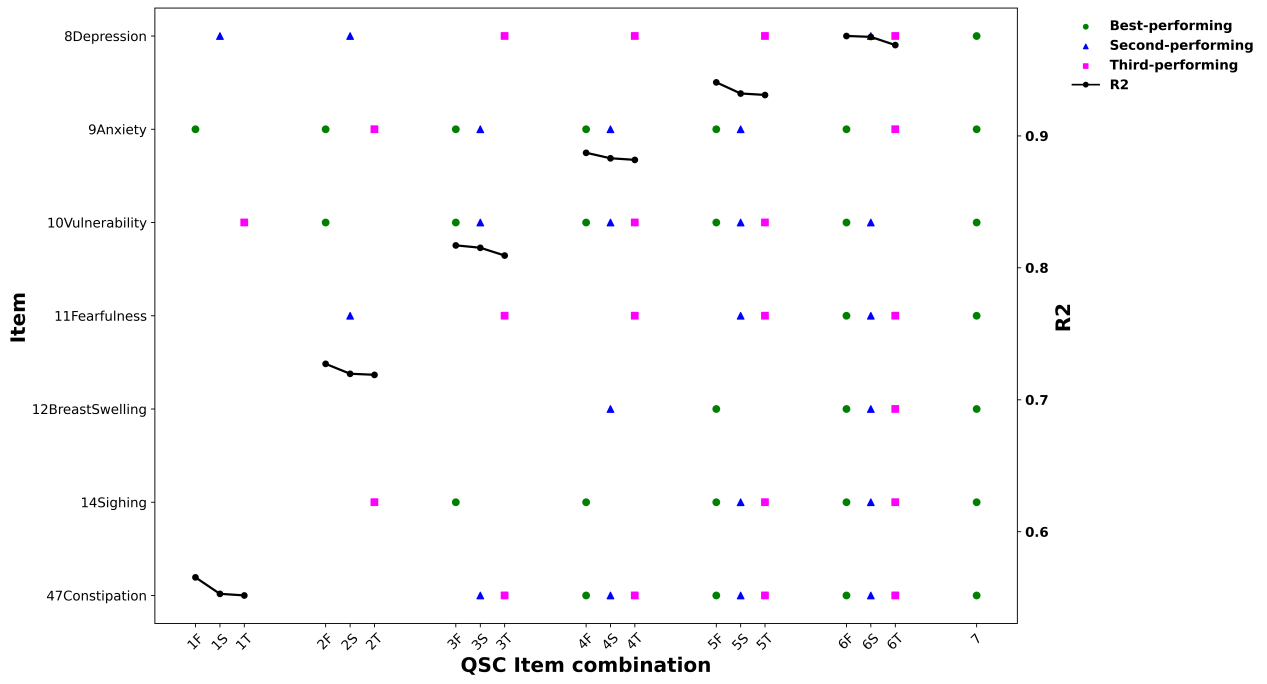


(H)


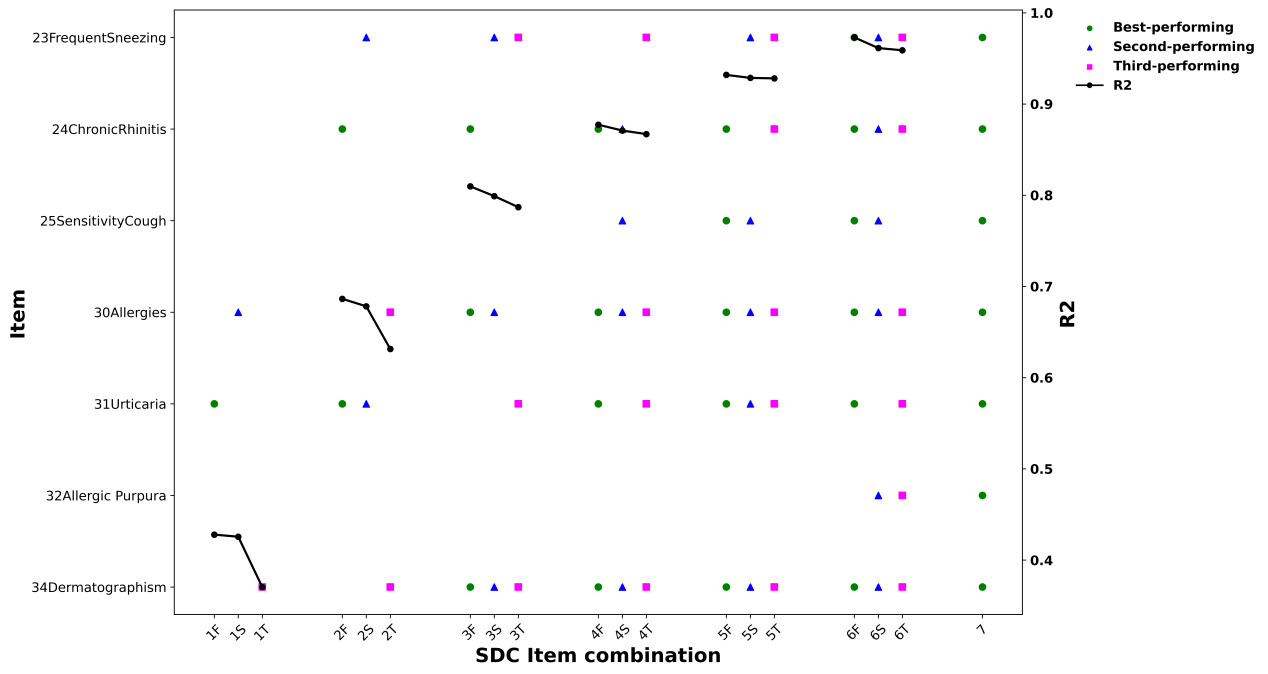


(I)

Figure S2 Top three item combinations and their corresponding R^2^ based on score results.

Note. The left vertical axis represents the specific item numbers and content included in the subscales of a particular body constitution, while the right vertical axis indicates the R^2^. The horizontal axis represents different combinations of items, with F denoting the best-performing combination, S the second best, and T the third best. The positions of scatter points with specific shapes and colors illustrate which items are included in item combinations of varying performance and item numbers, while the right vertical axis shows the R^2^ for these item combinations. A, B, C, D, E, F, G, H, and I represent the results for GTC, QDC, YaDC, YiDC, PDC, DHC, BSC, QSC, and SDC, respectively.
